# Tutorials on Machine Learning Tools for Simulations in Electrocatalysis

**DOI:** 10.1021/acsmaterialsau.5c00232

**Published:** 2026-08-05

**Authors:** Bruno Inácio da Silva Roux Leite, Anna Stepanova, Timmo-Hendrik Pukk, Denis Savchenko, Thor Kongstad Madsen, Nadezda Kongi, Vladislav Ivanistsev

**Affiliations:** † Institute of Chemistry, 37546 University of Tartu, Tartu 50411, Estonia; ‡ Department of Chemistry, 61769 University of Latvia, Jelgavas iela 1, LV-1004 Riga, Latvia

**Keywords:** Machine-learned interatomic potential, Bayesian statistics in optimization, Density Functional Theory, Molecular Dynamics, neural network-based exchange-correlation functional, deep learning-based models for calculations, Universal Model for Atoms, Message Passing Atomic Cluster Expansion

## Abstract

Machine learning (ML) is becoming an integral part of modern electrocatalysis, complementing and extending traditional density functional theory (DFT) simulations. Yet practical guidance on how these methods operate within realistic catalytic workflows remains limited. In this tutorial-style article we demonstrate seven ML approaches that accelerate or augment simulations relevant to the oxygen reduction reaction on platinum-based catalysts. These include machine-learned exchange–correlation functionals, Gaussian-process optimizers, Bayesian configuration search, and a range of ML interatomic potentials capable of geometry optimization and molecular dynamics at near-DFT accuracy. Each approach is illustrated on a chemically meaningful structural model and implemented in a reproducible Python notebook format. The tutorial highlights where ML currently provides clear benefit, where its accuracy is bounded by the underlying training data, and how these tools integrate with standard DFT workflows. Thus, the tutorial offers a compact and accessible introduction to usable ML tools for electrocatalysis and outlines the opportunities and practical limitations of their application in simulations of catalysts.

## Graphical abstract

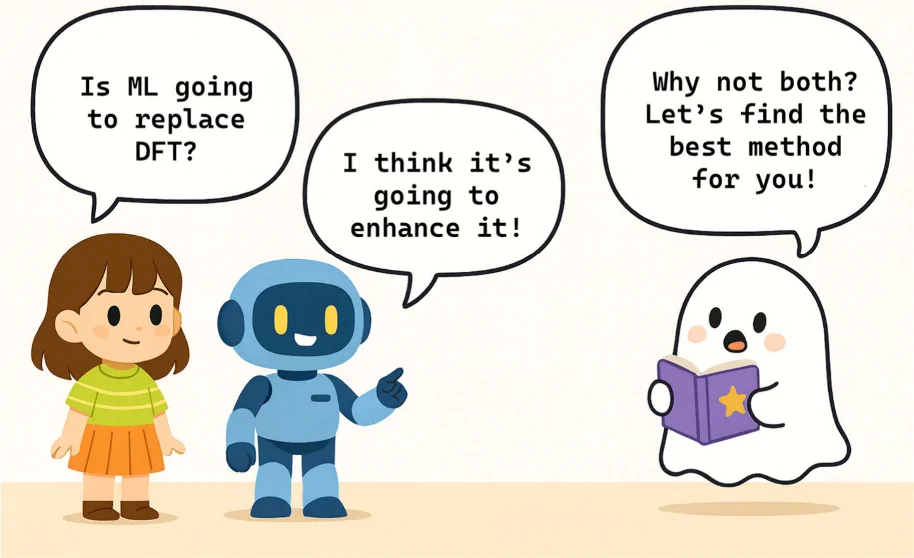

## Introduction

Machine Learning (ML) is becoming a part of computational electrocatalysis, where predictions traditionally relied on electronic-structure methods. For decades, density functional theory (DFT) has provided the main route to describe adsorption states’ structures, adsorption energies, and structure–activity trends.^1,2^ Despite its widespread use, accurate DFT modeling requires careful verification of computational settings, reproducible workflows, and awareness of the methodological approximations involved.^2–8^ Although the DFT approach is reliable, it is limited by its computational cost, which restricts the extent to which the chemical space of possible catalysts can be explored. If this can be solved by ML, it will allow large-scale screening of thousands of potential structures^9^ and enable the analysis of long-duration dynamics necessary to fully capture electrocatalytic reaction pathways. Accordingly, integration of ML causes a paradigm shift in computational electrocatalysis.^10^

This tutorial article illustrates the usage of ML approaches and tools in electrocatalysis. It consistently refers to three concepts: the approach, tool, and model. An approach defines a strategy for how ML interacts with DFT to accelerate, substitute, or extend it. A tool is the code that implements this strategy and can be executed and verified. A model is the chemical system used to demonstrate the idea, such as a Pt(111) surface (Figure 1) that serves as a reference in both computational and experimental electrocatalysis.

**Figure 1:**
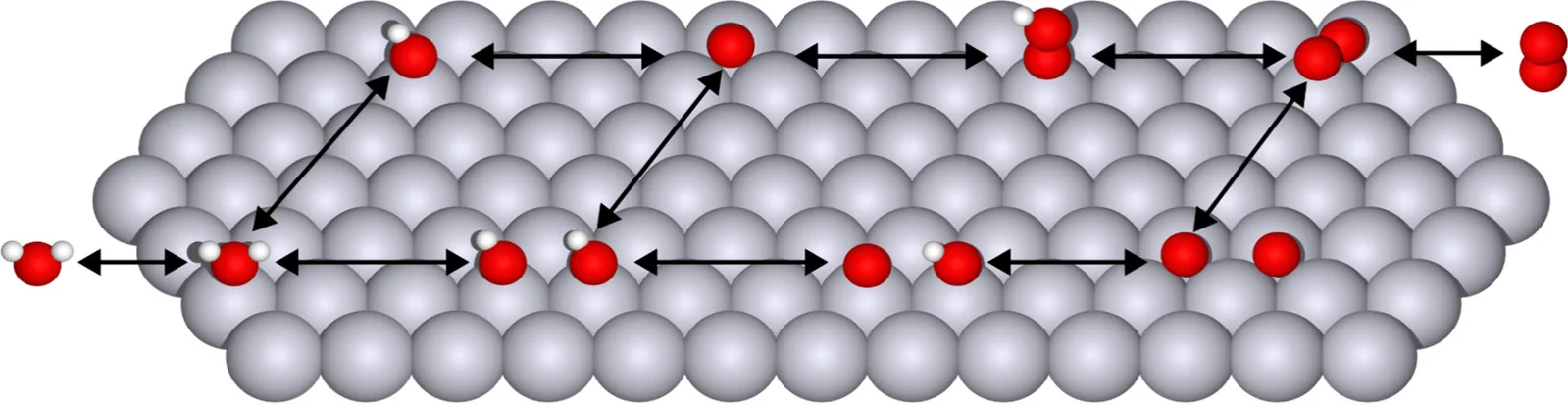
Illustration of an interface and mechanisms of oxygen electrocatalysis. From left to right for oxygen evolution reaction and from right to left for oxygen reduction reaction. The sequence illustrates the transformation from water to oxygen through surface-bound intermediates (O, OH, OOH) adsorbed on the Pt(111) surface.^11^

Below we focus on catalysts containing platinum because they define benchmarks for key electrochemical reactions, including the oxygen reduction reaction (ORR), as illustrated in Figure 1. The models were inspired by catalysts used for reactions involving carbon, nitrogen, and chlorine compounds.^12–16^ Each model is kept minimal but chemically sound, meaning that it preserves the correct coordination, bonding motifs, and surface terminations expected for the corresponding catalytic environment to allow direct interpretation and reproducibility of the results via Python Notebooks.^17^ All models used in the tutorials and figures are intentionally simplified for learning purposes and are not intended to represent production-level surface models. Quantitative research applications would require more realistic slab models, typically including several catalyst layers. The tutorial article is organized into seven notebook-based tutorials, each linking an ML approach with a representative catalytic model. All notebooks are provided in the Supporting Information (SI) and at the GitHub and Zenodo repositories, see the Data availability statement. These notebooks can be viewed and executed at mybinder.org. We tested the notebooks in the Google Colab environment,^18^ which allows anyone, even with low computer literacy and resources, to explore how ML methods integrate into standard computational workflows in electrocatalysis. We deliberately focused on approaches for which we found workable tools that can be installed, executed, and reproduced without modification.

A standard DFT workflow can be described as a sequence of steps, as illustrated in Figure 2. The calculation begins from a given atomic configuration. An initial electron density is used to construct the electronic description, including the effective potential.^19^ From this potential the Kohn–Sham equations are solved to obtain the electronic states; a new electron density is constructed and the total energy is evaluated. Once the convergence criteria are met, the calculation proceeds to force calculation. In geometry optimization, if the forces do not satisfy the convergence threshold, a position generator updates the atomic positions and the cycle restarts from the atomic configuration step; otherwise, the final output is obtained.^20^ In Molecular Dynamics (MD), forces are computed and used to update atomic positions and velocities at each time step; this cycle repeats until the desired simulation time is reached, and the output is the trajectory. The tutorials analyzed in this work correspond to different stages of this workflow, as illustrated in Figure 2: Tutorial 1 addresses electronic description and energy calculation, Tutorial 2 – positions generator, Tutorials 3 and 4 – atomic configuration, and Tutorials 5–7 – force calculation to run MD simulations.

**Figure 2:**
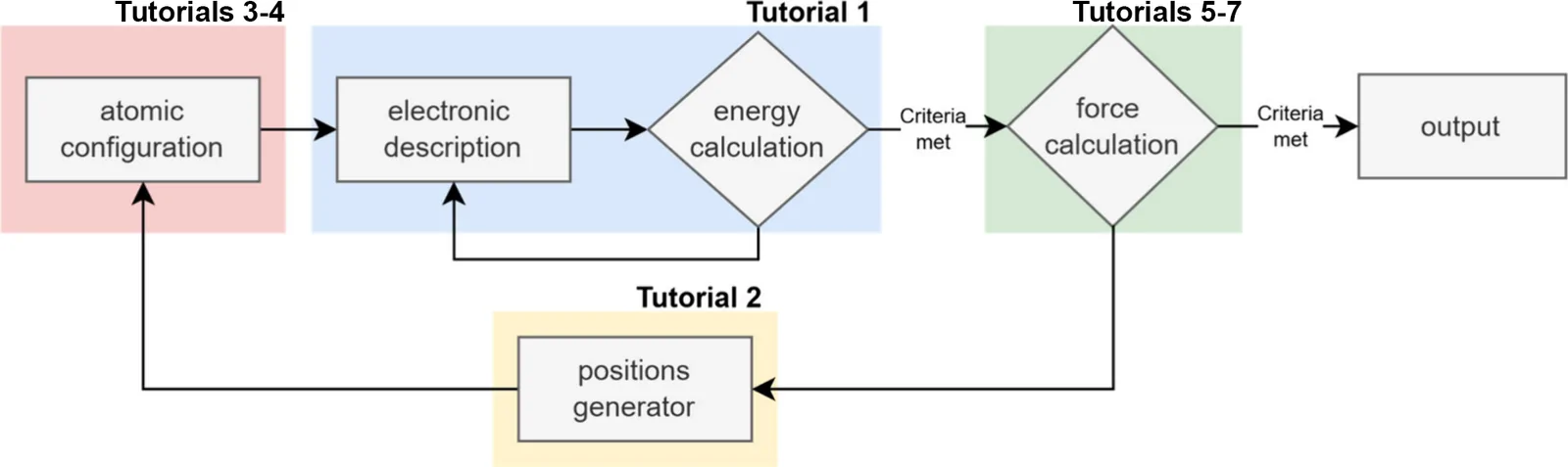
Schematic representation of a general computational workflow, including modules focused on atomic configuration, electronic description, energy calculation, force evaluation, and position updates when convergence criteria are not met. Colored boxes indicate the stages addressed by the tutorials: Tutorial 1 (electronic description and energy calculation), Tutorial 2 (position generator), Tutorials 3–4 (atomic configuration), and Tutorials 5–7 (force calculation).

## Tutorial 1. Machine-Learned Exchange–Correlation Functionals

### Approach

The standard DFT workflow can be described in three main parts: structural input, the self-consistent field cycle, and convergence analysis. Inside the self-consistent field cycle the electron density and related electronic quantities are obtained by solving the Kohn–Sham equations with an effective potential composed of the Hartree, external, and exchange-correlation (XC) contributions. The XC functional is approximated and introduces systematic inaccuracies in the electronic structure and related properties. This has motivated the development of new ML-based XC functionals designed to improve accuracy and transferability while maintaining low computational cost. Among them, the CIDER formalism demonstrates how nonlocal electronic effects can be represented within a data-driven framework while keeping the physical constraints of DFT. Another example of an ML-based XC functional is Skala,^21^ developed by Microsoft Research AI for Science. Although the current version, skala-1.1, is more suitable for molecular main-group chemistry than for transition-metal chemistry, the authors of the Skala paper intend to continue expanding the model’s training database, which may improve the model’s transferability. Overall, these ML XC functionals aim to improve upon conventional approximate XC functionals within their training range and may yield more reliable results for systems where nonlocality plays a major role.

### Notebook

In Tutorial 1 notebook, we applied a CIDER-type functional to perform a single-point calculation on a Pt@MnO_2_ model system, representing a platinum active site supported on a manganese dioxide surface relevant to catalytic applications.^22,23^ The electronic structure was evaluated by using the CIDER23X_NL_GGA functional for the geometry shown in Figure 3. For the purposes of the tutorial, we restrict the example to a single-point DFT calculation, as a full optimization would be too time-consuming to run within the tutorial setting while still demonstrating how ML XC functionals can be applied to periodic solid-state catalytic systems.

**Figure 3:**
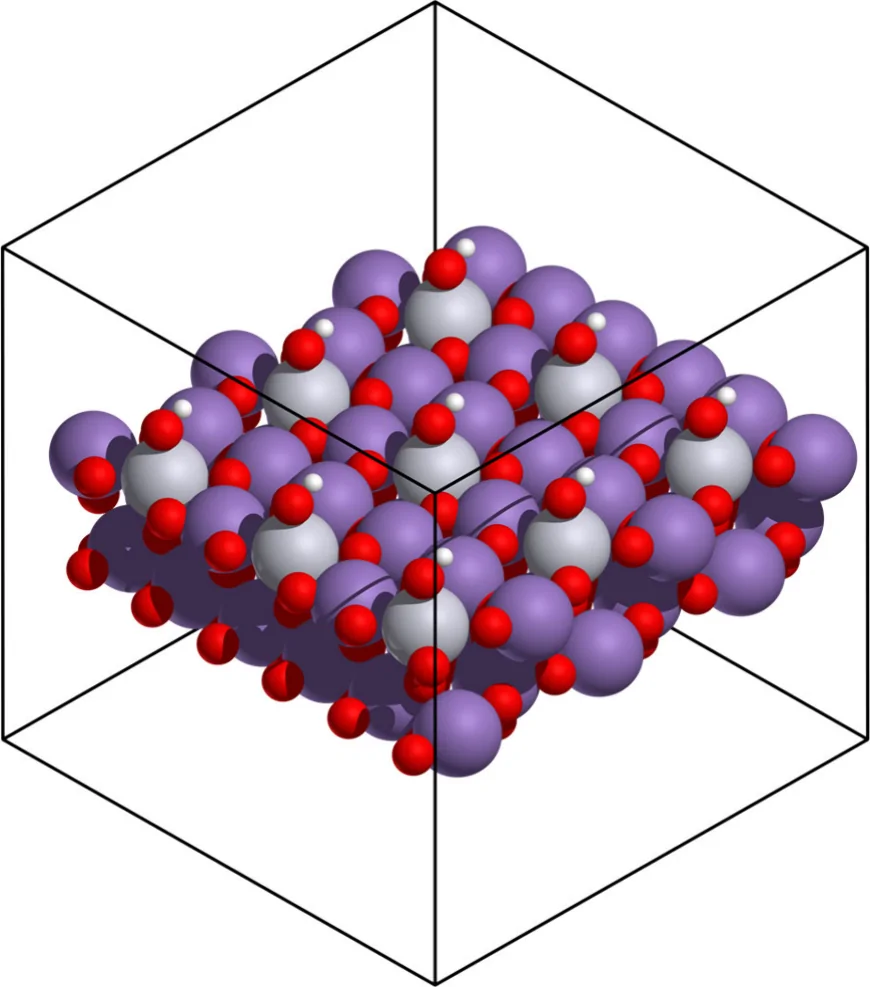
Geometry of a Pt@MnO_2_ used to perform single point calculation with the CIDER (Computationally Informed Density-Exchange Reconstruction) machine-learned exchange–correlation functional. The ML-enhanced XC functional improves the description of electronic interactions while preserving the computational efficiency of conventional DFT for solid-state catalytic systems.

### Applications

ML XC functionals, such as CIDER, can serve as drop-in replacements in conventional DFT functionals. Because analytical forces are available, they can also be used for geometry optimizations and molecular dynamics. They can also correct band gaps and resolve the CO puzzle, i.e., adsorption of CO on metal surfaces.^24^ The main challenge for ML density functionals is their transferability and generalization across chemical space, with the accuracy often dropping outside the training domain. This turns out from using the full electron density as input and from numerical instability in self-consistent DFT calculations.^25^ The progress depends on developing compact, transferable density features and stabilizing the self-consistent field cycle procedure. While the current models are still behind conventional DFT in accuracy, rapid advances suggest that broadly applicable ML functionals are within reach.^25^

## Tutorial 2. Gaussian Process Structure Optimization

### Approach

Building on the idea that DFT itself can be enhanced with ML, Tutorial 2 notebook shifts the focus from the electronic structure level to the structural level, using Gaussian process regression for geometry optimization. In electrocatalysis simulations, finding stable atomic structures often requires a sequence of DFT calculations, each of which is expensive on its own. Gaussian process regression offers a way to reduce the number of such calculations by learning the relation among atomic configurations, energies, and forces from a small initial set of calculations.^26^ For example, the GPMin algorithm applies this idea to geometry optimization.^27^ It builds a surrogate model of the energy surface and updates it as new DFT data are added. At every step, the model estimates both the energy and its uncertainty, guiding the next calculation to the most-informative configuration. In this way, GPMin replaces repetitive gradient-based searches with a data-driven path toward the lowest energy structure. However, GPMin does not always outperform gradient-based methods. In simpler systems, the initial training cycles can take up a large share of the optimization, offsetting the expected gains. Still, the method can be improved: GPMin has already been implemented into the nudged elastic band method and molecular conformer search algorithms.^28,29^

### Notebook

In the tutorial 2 notebook, we applied GPMin as implemented in GPAtom and ASE to a platinum graphitic carbon nitride catalyst (PtC6N8) with an OH intermediate on top to determine the most stable adsorption geometry.^27,30^ In essence, we performed a standard geometry optimization but replaced the usual Broyden–Fletcher–Goldfarb–Shanno algorithm with an ML alternative. This simple substitution illustrates how ML methods can directly enhance simulations still using DFT. The outcome of optimization is shown in Figure 4.

**Figure 4:**
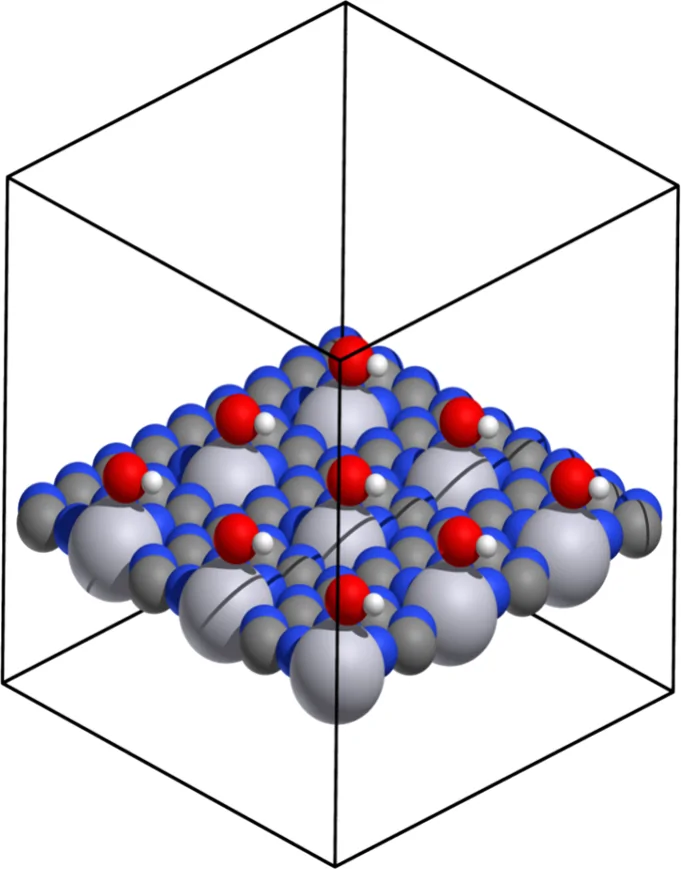
OH intermediate adsorption over a single-atom PtC_6_N_8_ catalyst after relaxation with the Gaussian-process-based GPMin optimizer. The Gaussian-process surrogate learns the potential-energy surface from DFT data, guiding geometry optimization toward the lowest-energy OH adsorption configuration.

### Applications

The GPMin method can serve as a general optimization tool for systems in which each DFT step is computationally expensive. In some cases, it reduces the number of steps required to locate structures with minimum energy.^27^ Similar Bayesian optimization strategies are implemented in related algorithms that apply the same principle to transition-state searches and reaction-path exploration. The method is particularly useful for adsorption studies, surface reconstruction, and cluster optimization, where it finds global energy minima with fewer total calculations.

## Tutorial 3. Bayesian Statistics in Search for Energy Extrema

### Approach

Mapping the full energy landscape of systems combining surfaces with adsorbents is one of the central challenges in DFT-based modeling of catalysis. Exploring all possible configurations of these fragments is rarely feasible, because each DFT evaluation is costly. To address this limitation, Bayesian optimization offers a statistical route to locate energy extrema efficiently.^31^ The Bayesian optimization structure search (BOSS) method applies this idea directly to simulations of catalytic interfaces.^32^ It constructs a probabilistic model of the energy surface from a small initial set of DFT results and updates it as new data are added. A Bayesian acquisition function then selects the next structure to test, balancing exploration of uncertain regions with refinement near low-energy configurations.^32^

### Notebook

In the Tutorial 3 notebook, we used BOSS to determine the preferred adsorption site of the OH intermediate on a PtN_4_-carbon single-atom catalyst. Several starting geometries are generated with OH placed at different orientations and distances from the Pt center. The BOSS reads obtained DFT energies and explores new configurations. In each step, the acquisition function proposes the next structure that combines low predicted energy with high uncertainty. The predicted configuration is almost on top of the expected site – the platinum atom, as shown in Figure 5.

**Figure 5:**
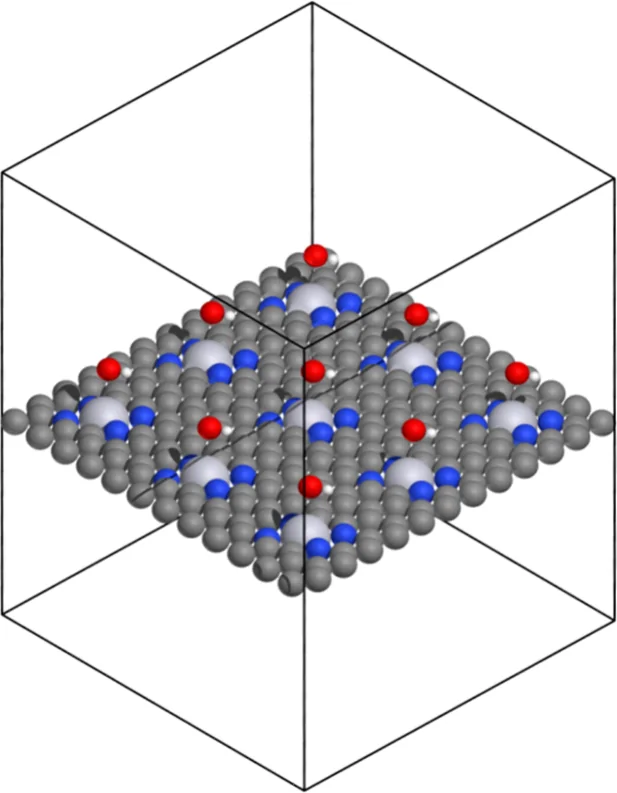
OH intermediate adsorption over a single-atom PtN_4_-carbon catalyst after relaxation with the BOSS (Bayesian Optimization Structure Search) method. This Bayesian-learning algorithm performs global exploration of the potential-energy surface, balancing uncertainty and predicted energy to identify the most stable adsorption geometry.

### Applications

The BOSS method is valuable for exploring adsorption structures, reaction intermediates, and surface rearrangements in catalytic systems composed of multiple fragments. It integrates naturally into DFT workflows to locate global or local minima efficiently. Moreover, it is coded in such a way that it can be fitted toward any math-based pure function problem. However, in our experience, the BOSS has limitations. An experienced electrochemist can often identify the active Pt site much more quickly by intuition, and convergence of the algorithm can be slow when the surface landscape is complex. We also encountered difficulties due to incomplete documentation, particularly of its Python application programming interface (API), and lack of recent maintenance. These aspects are common for many ML codes and reduce the practical reliability. Nevertheless, the benefit of using novel ML methods, such as a broader class of ML-driven optimizers, is high enough to discuss it further in the following section.

## Tutorial 4. Bayesian Statistics in Configuration Search

### Approach

Building on Bayesian exploration of adsorption geometries, tutorial 4 generalizes the approach to configurational complexity, enabling efficient searches over compositional ordering and atomic arrangements in multimetallic nanoparticles. A recurring challenge in computational electrocatalysis is identifying stable configurations in systems in which multiple metals compete for atomic positions. Traditional local optimization methods cannot easily capture the interplay between the composition and atomic ordering. Bayesian optimization provides a more global approach, allowing for efficient exploration of such complex landscapes.^31^ The BEACON (Bayesian Exploration of Atomic Configurations) method extends this principle by combining global exploration with Gaussian process modeling. It represents each configuration through a global structural fingerprint that captures both composition and geometry. This makes BEACON suitable for multimetallic nanoparticles, where the distribution of elements strongly influences the stability and catalytic performance.^33^

### Notebook

In the Tutorial 4 notebook, we applied BEACON as implemented in GPAtom and ASE to a 13-atom Ag–Pd–Pt–Ru nanoparticle to examine its dealloying behavior and identify the most stable atomic arrangement.^27,30^ The chosen number of atoms Ag_3_Pd_2_Pt_3_Ru_5_ mimics the composition of an alloy predicted to be most active for ORR among Ag–Pd–Pt–Ru alloys.^34^ At each step, BEACON generates a new random configuration and calculates the energy with the chosen calculator object. The total energy and atomic configuration is then used to train the initial Gaussian process model, where each result is interpreted as a vectorised fingerprint in a surrogate space, which can be evaluated for the energy quicker than the actual coordinate space of the atoms with DFT. Within this surrogate space, test structures are then generated and optimized with the use of higher order spatial dimensions; ghost atoms with a fractional presence and atoms are also allowed to exchange identities. As the search progresses and is moved back into a real space, the model converges toward low-energy structures. As an illustration we put one of the resulting nanoparticles on top of carbon support in Figure 6.

**Figure 6:**
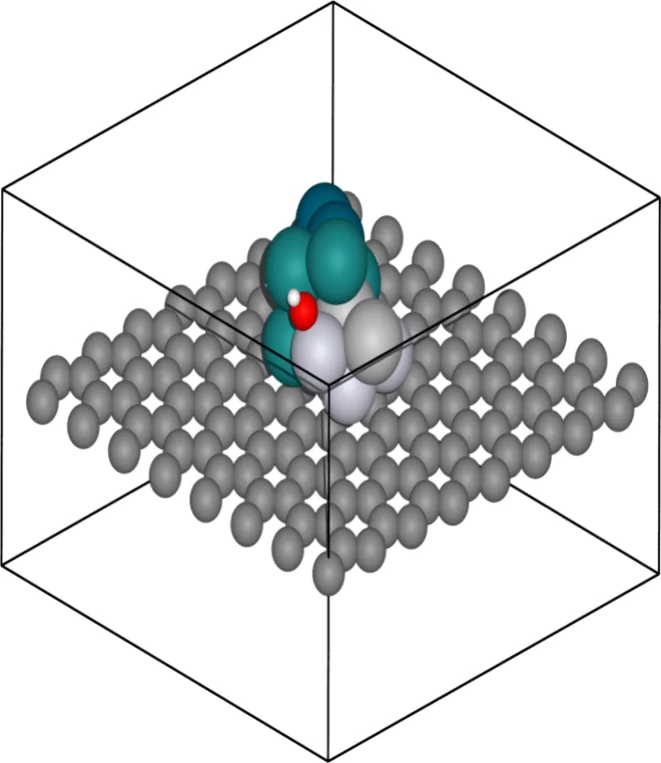
OH intermediate adsorption at the Ag_3_Pd_2_Pt_3_Ru_5_ nanoparticle catalyst identified using BEACON and placed over a carbon support. This ML approach applies global Bayesian optimization with Gaussian-process modeling, and it captures alloy rearrangement and segregation behavior in multimetal nanoparticles.

### Applications

The BEACON method enables global optimization of multimetal nanoparticles by locating low-energy configurations across broad compositional and structural spaces. It is particularly useful for studying core–shell formation, surface segregation, and elemental ordering in alloy catalysts. Similar ML-based optimizers follow related principles. In our experience, BEACON offers an efficient way to explore alloy stability, but its setup can be demanding and convergence may depend on the choice of kernel or initial training data. Beacon can also be modified into other geometric problems by writing a new random structure generator; this enables the calculations of solvent configurations or conform searching.

## Tutorial 5. ML Interatomic Potentials for Simulations of Catalytic Surfaces

### Approach

Moving from DFT-driven structural exploration toward partial DFT calculations replacement, Tutorial 5 introduces pretrained ML interatomic potentials. In simulations of catalytic surfaces, the computational cost of DFT limits the accessible system size and time scale. ML interatomic potentials address this limitation by reproducing DFT-level energies and forces at much lower cost.^35^ These models achieve this by learning the relation between atomic configurations and the corresponding energies and forces from large data sets of DFT calculations. The Universal Model for Atoms (UMA),^36–38^ developed within Meta’s Open Catalyst Project (OCP),^39^ represents a recent generation of such models. The first data sets OC20 ^35^ and OC22 ^40^ covered DFT calculations of adsorbates on catalyst surfaces across diverse materials – including metals, alloys, oxides, carbides, and other multicomponent compounds – and covered adsorption, diffusion, and surface reaction processes relevant to heterogeneous catalysis. Most recent data sets OC25 and OMol25 include adsorbate–surface interactions and solvent environments, enabling ML potentials to better capture catalytic systems under more realistic conditions.^41,42^ Specific models were trained on these data sets to maintain DFT consistency within provided chemical spaces. The key advantage of UMA is its speed. In comparison to DFT, it accelerates structure optimization and screening by orders of magnitude while retaining the accuracy of its training data.

### Notebook

In Tutorial 5 notebook, we used UMA to model a Pt(111) surface with OH groups at coverage of 1/4. The pretrained model was accessed through the OCP interface and employed to calculate forces and energies to reach the optimal geometry shown in Figure 7. The procedure followed a standard DFT workflow, yet with ML interatomic potentials of UMA yielding the optimized surface configuration and adsorption energy in a fraction of the usual DFT computational time.

**Figure 7:**
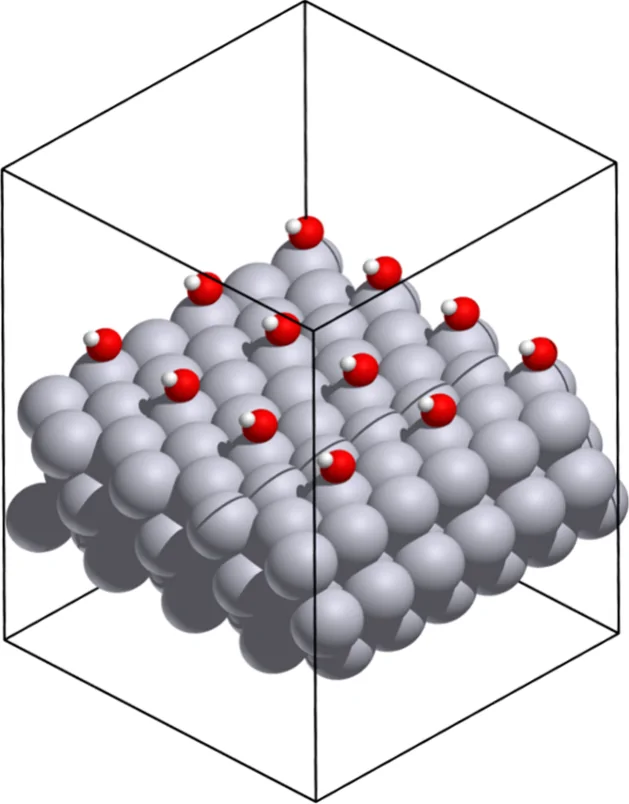
Pt­(111) surface with OH groups at a coverage of 14 , optimized using UMA ML interatomic potentials. This ML-based simulation employs pretrained universal materials models to predict adsorption geometry and surface relaxation, efficiently reproducing DFT-level accuracy in the optimized structure while significantly reducing computational cost.

### Applications

UMA and related ML interatomic potentials are effective for screening adsorption geometries, reaction intermediates, and surface reconstructions. They are particularly useful for systems resembling their training data, such as metal surfaces with small adsorbates. Their strength lies in computational speed and reproducibility. The process works as follows: The system first generates dozens of different starting configurations for the adsorbate on the catalyst’s surface. It then performs a local optimization, for each configuration to find its most stable state. This relaxation is driven by model-predicted forces provided by the ML, such as GemNet-OC or EquiformerV2.^36–38^ However, such models do not yet capture solvent effects, charge transfer, or large-scale restructuring. Hybrid models such as SpookyNet extend this approach by incorporating explicit electronic and nonlocal effects, narrowing the gap between DFT and ML-based simulations.

## Tutorial 6. ML Force Fields to Simulate a Realistic Pt–OH–H_2_O Interface

### Approach

Building on pretrained ML interatomic potentials optimized for fast, DFT-consistent surface relaxations, tutorial 6 introduces SpookyNet, a hybrid ML force field designed to overcome the limitations of classical force fields,^43^ while classical force fields are widely used to simulate large systems, but they cannot describe bond formation, charge redistribution, or electronic polarization. In contrast, DFT provides an accurate description of these effects but at a high computational cost.^44^ ML force fields bridge this gap by learning energies and forces from DFT data and predicting them much more quickly.^45^ In practice, these models learn the potential energy surface directly from atomic configurations, enabling a flexible description of bonding environments beyond the limitations of fixed analytical force fields. The SpookyNet model represents a new generation of ML force fields that includes electronic degrees of freedom and nonlocal interactions through a self-attention mechanism. This design allows it to capture quantum effects, such as charge transfer and long-range polarization, that are inaccessible to conventional ML interatomic potentials.

### Notebook

In the Tutorial 6 notebook, we used SpookyNet to simulate a realistic Pt–OH–H_2_O interface. This system is iconic for research of ORR on Pt(111) as it is associated with characteristic cyclic voltammetry peak and can be visualized with scanning tunneling.^46,47^ We constrained the Pt atoms to their bulk positions and allowed the adsorbate and solvent to relax. SpookyNet predicted energies and interatomic forces. The optimized structure in Figure 8 reveals hydrogen bonding between water molecules and the OH intermediate on the metal surface, which reproduces the expected interfacial behavior.

**Figure 8:**
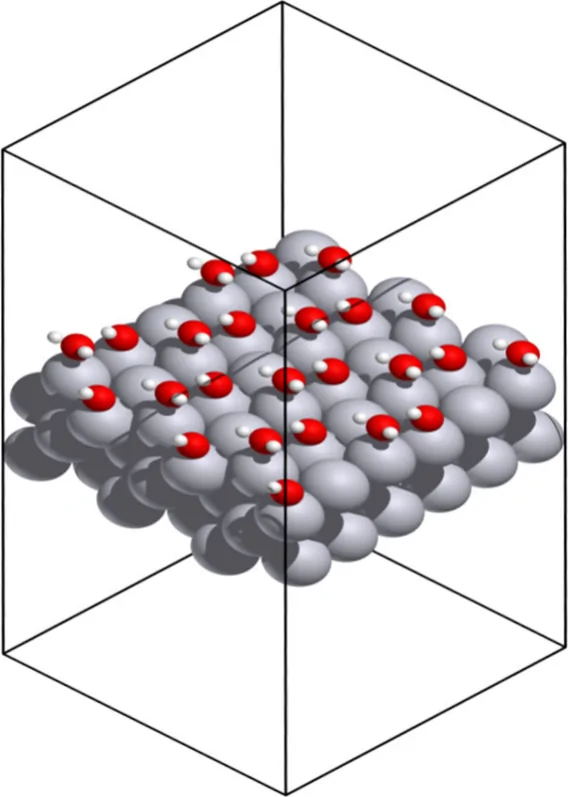
Pt­(111)–OH–H_2_O interface optimized with SpookyNet. This hybrid machine-learning force field combines neural-network potentials with explicit physical interactions to account for polarization and long-range electronic effects at catalytic interfaces.

### Applications

ML force fields with electronic terms, such as SpookyNet, extend simulations to systems in which polarization and charge transfer play key roles. They can be used for the geometry optimization, molecular dynamics, and vibrational analysis of complex materials. Their accuracy depends on the quality and diversity of the training data,^45^ but their efficiency is comparable to that of classical force fields. While computationally heavier than simpler ML interatomic potentials, models like SpookyNet make it possible to explore electrochemical interfaces with near-DFT accuracy at a manageable cost.

## Tutorial 7. Machine-Learning Interatomic Potentials for Molecular Dynamics

### Approach

Tutorial 6 focuses on static or locally optimized interfacial structures using ML force fields with explicit electronic degrees of freedom, Tutorial 7 advances to large-scale molecular dynamics using MACE, which emphasizes efficient many-body message passing to access long-time- and length-scale interfacial dynamics at near-DFT accuracy. Molecular dynamics (MD) simulations are essential for understanding the structure and motion of atoms at catalytic interfaces. However, DFT-based MD is computationally expensive and limited to short time scales. ML interatomic potentials address this limitation by learning energies and forces from DFT data and predicting them at near-classical MD speed.^48^ In these models, atomic structures are represented through descriptors of the local chemical environment, allowing the ML model to learn the potential-energy surface while preserving the symmetries of the atomic system. The MACE (message passing atomic cluster expansion) framework combines the physics of cluster expansions with message-passing neural networks, enabling efficient treatment of many-body interactions and chemical environments. Its purpose is to reproduce DFT-level accuracy for large systems and long trajectories without the prohibitive cost of DFT calculations. For MD simulations, ML interatomic potentials appeared as a viable alternative to the force fields. One promising direction is improving the message passing from traditional two-body to many-body, such as with atomic cluster expansion.

It should be noted that, like most MLIP-, FF- and DFT-based MD simulations, Tutorial 7 operates at constant surface charge rather than constant potential mode. It is in principle possible to transform one into the other by running a vast amount of calculations and applying Legendre transform like in the Generalized computational hydrogen electrode (GCHE) framework.^49^ Direct MLIP-, FF- and DFT-based constant potential methods (CPM) already exist.^50–52^ As of 2026, users aiming to quantitatively compare experimental and computational data under applied potential should be aware of a current limitation: most simulations are run using constant surface charge methods rather than constant electrode potential. Existing CPM differ in core principles and remain at an early adoption stage. In the future, the use of MLIP can facilitate adoption of both direct CPM as well as GCHE approaches.

### Notebook

In the Tutorial 7 notebook, we used MACE to perform MD simulations of a Pt(111)–OH–water interface,^53^ representing a realistic electrochemical environment for oxygen reduction. We use the model with three layered Pt(111) slab and 32 water molecules, thus demonstrating the dynamics of water near the catalytic surface.^54^ The model calculated interatomic forces and energies on the fly, allowing us to follow the motion of atoms in time. The trajectory shows how water molecules organize around the surface and interact with hydroxyl groups through hydrogen bonding. Over time, the liquid layer continuously rearranges, forming and breaking hydrogen bonds at the interface. This simulation illustrates the dynamic behavior of interfacial water and the stability of adsorbed OH on the metal surface, as shown in Figure 9.

**Figure 9:**
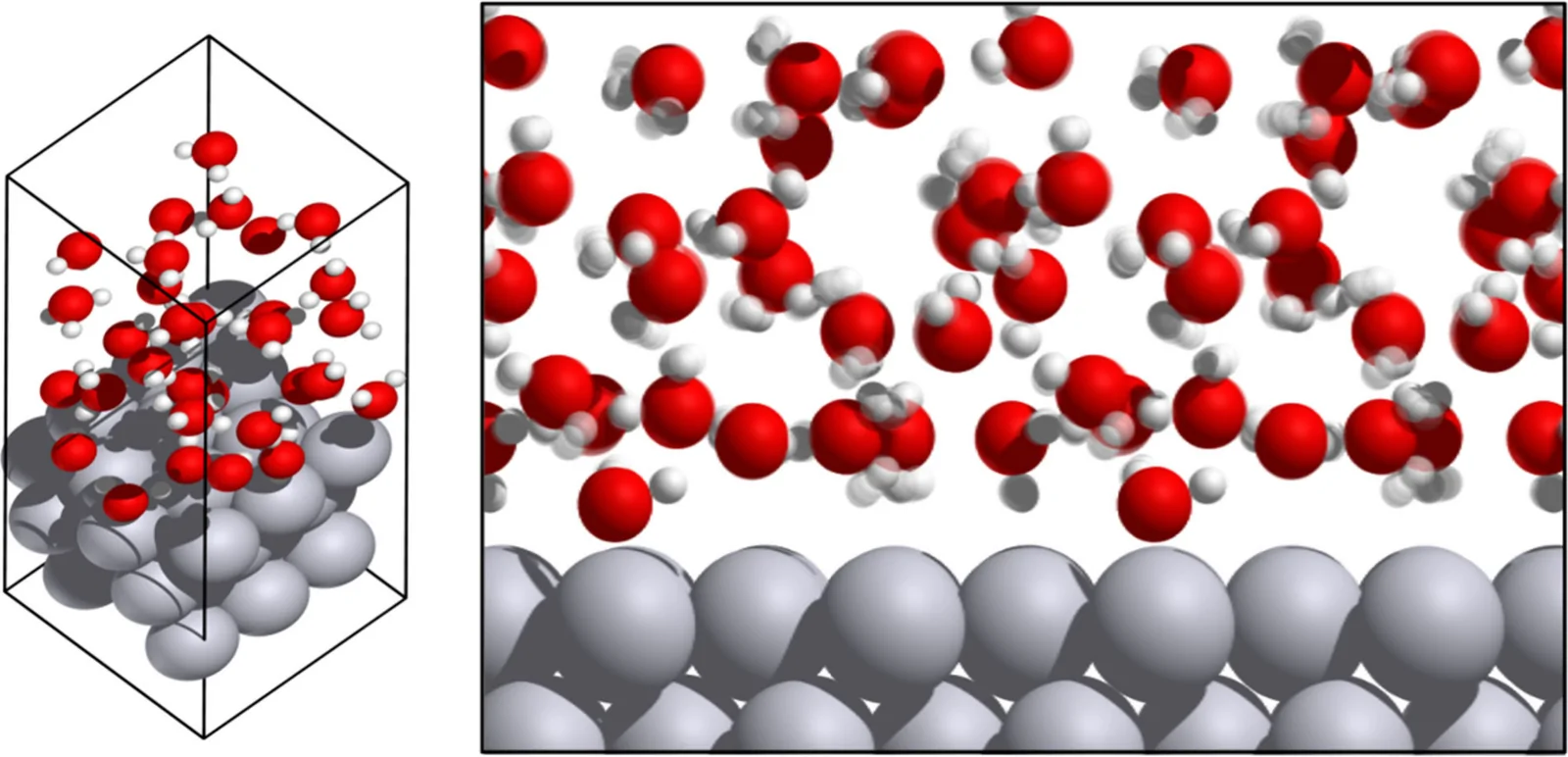
Dynamic behavior of water molecules at a Pt(111)–OH–water interface simulated with MACE (Message-Passing Atomic Cluster Expansion). On the left, the full image of the structure, and on the right, an overlap of frames from the MD simulation, to illustrate the natural movement of the water.

### Applications

MACE and related ML tools extend atomistic simulations to time and length scales inaccessible by DFT. They are suited for studying adsorption dynamics, solvation effects, and structural changes at interfaces. This extends to diverse applications, including the relaxation of surface states of varying type and size.^55^ Their applicability spans diverse areas, from the detailed analysis of adsorbate–adsorbate interactions,^56^ and the exploration of transition states to capture reaction pathways, for example in microkinetic models of CO_2_ reduction.^57,58^ Models such as VASP-MLFF follow similar principles.^59^ Although these models rely on the quality of their training data and may lose transferability outside that range, they provide a practical route to explore realistic catalytic environments with DFT-level detail.

## Machine-Learning Potentials

The most rapidly developing methods among recent studies are machine learning interatomic potentials (MLIPs), which have become one of the most active and widely applied approaches in atomistic simulations. For this reason, we focus on them in greater detail in this section.

To clarify the differences between the machine-learning potentials discussed in Tutorials 5–7, Table 1 summarizes their main characteristics and typical applications. UMA, SpookyNet, and MACE all aim to reproduce DFT-level energies and forces at reduced computational cost; they differ in their design goals, accessible system sizes, and the types of interactions they describe. These differences determine the kinds of problems for which each approach is most suitable, ranging from rapid screening of adsorption geometries to reactive simulations with electronic effects and large-scale molecular dynamics of catalytic interfaces.^36–39,43,48,55^ Presenting these models side-by-side helps position them within the broader computational workflow and highlights how ML potentials can be selected depending on the desired balance between accuracy, system size, and simulation time scale.^60^

**Table 1:** Comparison of Representative MLIP Potentials^a^

Software	Main purpose	System size	Time scale	Interactions	Typical use
**UMA**	Fast structure relaxation and screening	Small to large systems	Geometry optimization and short MD	Short-range interactions	Fast evaluation and relaxation of atomistic structures^61,62^
SpookyNet	Reactive simulations with electronic effects	Small to large systems	Geometry optimization and short MD (ps)	Short-range and long-range electrostatics	Fast evaluation and relaxation of atomistic structures^43,63,64^
**MACE**	Large-scale molecular dynamics	Medium to large systems	Geometry optimization and MD up to tens of ns	Short-range many-body	MD of surfaces, interfaces, and bulk systems^48,65^

a The table summarizes the primary purpose, typical system size, accessible time scale, types of interactions captured, and common applications for UMA, SpookyNet, and MACE models in computational catalysis and materials simulations.

Despite their complementary strengths, UMA and MACE share a fundamental limitation: they rely on local atomic environments within a fixed cutoff radius and cannot globally equilibrate chemical potential. When charge availability controls binding energetics these models can produce unphysical results without any warning from standard accuracy metrics. Recent benchmarking of short-range MLIPs at charged metal/electrolyte interfaces has shown that models can exhibit low energy errors while yielding incorrect water orientation and ion distributions, because the global surface charge lies outside the descriptors’ receptive field.^60^ Users applying the workflows in Tutorials 5–7 to systems beyond well-screened metallic surfaces should therefore validate MLIP predictions against DFT reference calculations before drawing physical conclusions.

A related limitation is that of the training objective. Fourth-generation potentials and charge equilibration architectures exist to address long-range charge redistribution explicitly,^66^ and SpookyNet incorporates global electronic embeddings toward this end.^43^ However, models such as UMA and MACE were trained solely on total energies and forces without charge partitioning in the loss function. They cannot reliably capture shifting oxidation states during redox events, regardless of the architectural capacity. The recently released OC25 and OMol25 data sets include partial charge information,^41,42^ but until charge-aware foundational models are broadly validated, users modeling systems with active redox chemistry should apply these workflows with caution.

## Concluding Remarks

We wrote this Tutorial with two simple goals: (1) to identify ML tools that work out of the box and (2) to examine how far they can take standard simulations. This required choosing methods that install without friction, run as documented, and give results that could be verified. Computational electrocatalysis offers many ideas but fewer stable implementations; therefore, the focus stayed on approaches that operate reliably. The notebooks developed for this Tutorial article can support seminar-style learning, because each Tutorial notebook provides a complete, reproducible workflow that readers can follow at their own pace. Apparently, these are mainly ML-enhanced DFT, adaptive optimizers, Bayesian search strategies, and interatomic potentials that are already able to extend the accessible length and time scales of typical computational workflows, as illustrated in Figure 2.

During the preparation of the notebooks, steady progress in the field became evident, together with the fragile nature of many software packages. Several tools that were functional a year ago are now unsupported, such as with OCP and FAIRChem. Code evolves quickly and sometimes outpaces its documentation, such as with BOSS. Access restrictions add friction that complicates systematic testing, such as with UMA. These issues do not reduce the scientific usefulness of novel ML methods but highlight the practical hurdles that still exist.

Even so, the direction is encouraging. ML interatomic potentials already deliver DFT-consistent energies and forces at speeds that change what can be explored. ML density functionals, such as CIDER, pursue improved physics through nonlocal terms. Bayesian tools help explore configurational space in a way that often feels more efficient than traditional searches. ML force fields enable running simulations on a laptop, while their DFT analogues require a supercomputer.

Our tutorial notebooks are structured in a way that allows both researchers and seminar participants to reuse them as self-contained demonstrations. They intend to show that new research opportunities have opened. We hope that the provided tutorials will help the readers to capture what is feasible today and point toward developments that may soon become everyday practice in electrocatalysis. This makes the notebooks suitable for both research workflows and use in seminars or group training sessions.

As the last note, let us stress that while the focus of this tutorial article is electrocatalysis, many of the ML methods discussed here are equally relevant to other areas of surface and materials science, including heterogeneous catalysis, photocatalysis, and semiconductor interface modeling.^67–69^

## Supplementary Material



## Data Availability

The data and code required to reproduce the analyses reported in this article are openly available at GitHub https://github.com/doublelayer/ACSMaterialsAu_2025_ML-modeling and Zenodo 10.5281/zenodo.20432908.

## Notes

Seven Python notebooks, each corresponding to one of the tutorial sections described in this article. The notebooks implement the following workflows: (1) single-point DFT calculation using the CIDER machine-learned exchange–correlation functional on a Pt@MnO_2_ system; (2) geometry optimization of OH adsorption on a PtC_6_N_8_ catalyst using the Gaussian-process-based GPMin optimizer; (3) Bayesian adsorption-site search on a PtN_4_-carbon catalyst using BOSS; (4) global configuration optimization of an Ag_3_Pd_2_Pt_3_Ru_5_ nanoparticle using BEACON; (5) structure relaxation of an OH-covered Pt(111) surface using UMA ML interatomic potentials; (6) geometry optimization of a Pt(111)–OH–H_2_O interface using the SpookyNet force field; and (7) molecular dynamics simulation of a Pt(111)–OH–water interface using MACE (PDF)
