# Supplementary material for: Tutorials on Machine Learning Tools for Simulations in Electrocatalysis
Source: ACS Mater Au. 2026 Aug 5;6(6):1301–11. doi: 10.1021/acsmaterialsau.5c00232 (PMC13760637; doi:10.1021/acsmaterialsau.5c00232)
Supplement: Supplementary file 1 [file mg5c00232_si_001.pdf]

Supporting Information

for

Tutorials on Machine Learning Tools for Simulations in Electrocatalysis

Bruno Inácio da Silva Roux Leite,[a] *Anna Stepanova,*[b] *Timmo-Hendrik Pukk,*[a] *Denis Savchenko,*[a] *Thor Kongstad Madsen,*[a*] *Nadezda Kongi,*[a*] *Vladislav Ivanistsev.*[b*]

[a] Institute of Chemistry, University of Tartu, Tartu 50411, Estonia

[b] Department of Chemistry, University of Latvia, Jelgavas iela 1, LV-1004 Riga, Latvia

* thor.kongstad.madsen@ut.ee, nadezda.kongi@ut.ee, vladislav.ivanistsev@lu.lv

---

Supporting Information

The data and code supporting this work are publicly available. Tutorial-style Jupyter notebooks demonstrating the seven ML approaches described in the article are provided in the GitHub repository at: https://github.com/doublelayer/ACSMaterialsAu_2025_ML-modeling, and archived on Zenodo at https://doi.org/10.5281/zenodo.20432908. The repository includes environment configuration files (apt.txt, environment.yaml, postBuild) enabling reproducible execution via mybinder.org or locally.

The Supporting Information contains seven Colab notebooks, each corresponding to one of the tutorial sections described in this article. These Colab notebooks implement the following workflows: (1) single-point DFT calculation using the CIDER machine-learned exchange–correlation functional on a $Pt@MnO_2$ system; (2) geometry optimization of OH adsorption on a PtC_6N_8 catalyst using the Gaussian-process-based GPMin optimizer; (3) Bayesian adsorption-site search on a PtN_4-carbon catalyst using BOSS; (4) global configuration optimization of an $Ag_3Pd_2Pt_3Ru_5$ nanoparticle using BEACON; (5) structure relaxation of an OH-covered Pt(111) surface using UMA ML interatomic potentials; (6) geometry optimization of a Pt(111)–OH–H_2O interface using the SpookyNet force field; and (7) molecular dynamics simulation of a Pt(111)–OH–water interface using MACE. The Colab notebooks differ from the refined Python notebooks, and are meant to be run at Google Colab nodes online.

UMA license

The UMA model is distributed under the FAIR Chemistry License by Meta, available at https://huggingface.co/facebook/UMA/blob/main/LICENSE. To use the model in your own environment, go to Hugging Face (huggingface.co), create an account, and generate an access token under Settings > Access Tokens. Since tokens are personal and should not be shared, the tutorial notebook the model file is provided directly in the notebook environment.

Tutorial 1. Machine-learned exchange–correlation functionals

Goal of the notebook

The goal of this notebook is to provide step-by-step instructions on how to run ciderpress XC functionals. We do that by running a single point calculation on a TBLite optimized β-MnO2-Pt surface with an adsorbed OH.

Description of the method

The CIDER framework incorporates machine learning into density functional theory by constructing exchange–correlation (XC) functionals from descriptors derived from the electron density. Implemented through ciderpress and compatible with packages such as PySCF, these ML-based functionals can be used within standard DFT workflows to improve the description of electronic interactions while maintaining the computational efficiency of conventional functionals.

Ciderpress github: https://github.com/mir-group/CiderPress

Ciderpress webpage: https://mir-group.github.io/CiderPress/

Ciderpress article: Bystrom, K.; Kozinsky, B. Nonlocal Machine-Learned Exchange Functional for Molecules and Solids. Phys. Rev. B 2024, 110, 075130. https://doi.org/10.1103/PhysRevB.110.075130

Outline of theWorkflow

STEP 1. β-MnO2-Pt surface creation and relaxation (TBLite)

STEP 2. Surface + adsorbate relaxation (TBLite)

STEP 3. Single-point calculation (Ciderpress)

Installation of packages

First things first, we need to install the packages and import the neccessary functions. We need ASE, TBLite, Ciderpress and its functionals, and GPAW (MixerFull functional).

```
##################
##-installation-##
##################

%%capture

##-ASE
!pip install ase

##-TBLite
!pip install tblite==0.4.0

##-Ciderpress
!apt install libxc-dev
!pip install ciderpress

!wget 'https://zenodo.org/api/records/13336814/files-archive' -O cider_func
!unzip cider_functionals.zip

##-GPAW
!pip install gpaw
!echo "y" | gpaw install-data /usr/local/pkgs/gpaw-data-0.9.20000/
```

Next we import the necessary packages:

ASE https://ase-lib.org/

TBLite https://github.com/tblite

GPAW https://gpaw.readthedocs.io/

Ciderpress https://github.com/mir-group/CiderPress/tree/main/ciderpress

```
##################
##-dependencies-##
##################

##-ASE
from ase import Atom, Atoms
from ase.build import molecule, surface, add_adsorbate
from ase.io import read, write
from ase.visualize import view
from ase.optimize import BFGS

##-TBLite
from tblite.ase import TBLite

##-Ciderpress
from ciderpress.gpaw.calculator import CiderGPAW, get_cider_functional

##-GPAW
```

```
from gpaw import GPAW, MixerFull #-also needed for Ciderpress

```

STEP 1. β-MnO2-Pt surface

Here we download the unit cell for β-MnO2 and turn it into a surface. Using the ASE surface function we create a β-MnO2(110) slab and replace one of the surface manganese atoms with a platinum atom, creating the β-MnO2-Pt surface. After succesfully creating the surface, we use TBLite calculator and BFGS optimizer for relaxing the surface. For simplicity sake we assume charge = 0, multiplicity = 1, and we don't use spin_polarization.

```
#################
##-MnO2 object-##
#################

##-Get the initial .cif file from materials project:
!wget https://raw.githubusercontent.com/doublelayer/test_models/refs/heads/
mno2 = read('MnO2_beta.cif')

view(mno2, viewer='x3d')
```

```
--2026-05-22 11:56:47--  https://raw.githubusercontent.com/doublelayer/test_mode
Resolving raw.githubusercontent.com (raw.githubusercontent.com)... 185.199.111.1
Connecting to raw.githubusercontent.com (raw.githubusercontent.com)|185.199.111.
HTTP request sent, awaiting response... 200 OK
Length: 1045 (1.0K) [text/plain]
Saving to: ‘MnO2_beta.cif’

MnO2_beta.cif       100%[===================>]   1.02K  --.-KB/s    in 0s

2026-05-22 11:56:47 (49.4 MB/s) - ‘MnO2_beta.cif’ saved [1045/1045]
```

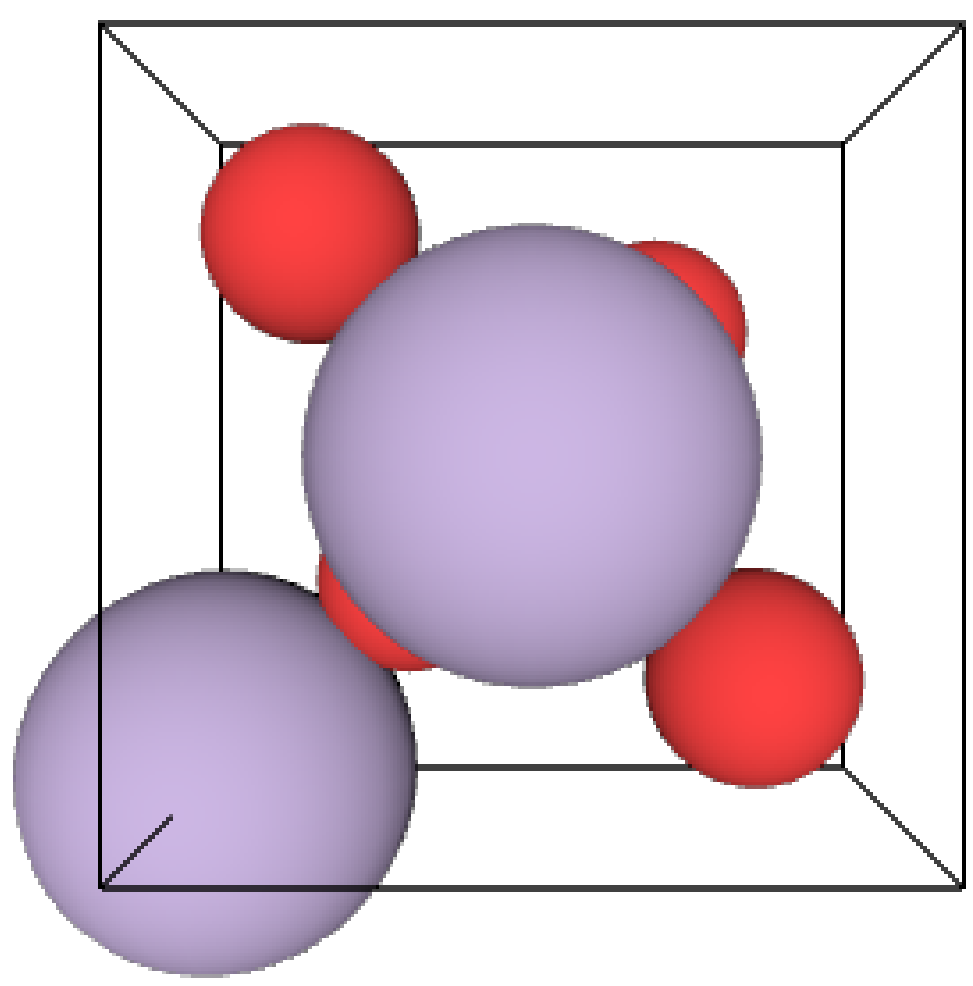

```
####################
##-MnO2-Pt object-##
####################

##-Creating the surface
slab = surface(mno2, (1,1,0), layers=2)
slab = slab.repeat((1,2,1))

##-Using the wrap() function to move topmost oxygens to the bottom
z = slab.positions[[a.index for a in slab if a.symbol == "Mn"], 2]
dz = (abs(z.max() - z.min()))
slab.cell[2,2] = 2*dz
slab.set_cell(slab.cell)
slab.positions[:,2] += 0.9*dz
slab.pbc=(True,True,True)
slab.wrap()

##-Replacing one of the manganese atoms with a platinum atom
slab += Atom("Pt", slab.positions[18])
del slab[18]

##-Setting the surface periodic in x and y coordinates
```

```
slab.pbc=(True,True,False)
slab.center(axis=2, vacuum=1.5*dz)
slab.positions[:,2] += -dz

view(slab, viewer='x3d')
```

Show hidden output

```
####################################
##-optimization of MnO2-Pt(1,1,0)-##
####################################

slab.calc = TBLite(
    method='GFN1-xTB',
    charge=0,
    multiplicity=1,
    spin_polarization=0,
    max_iterations=350,
)

opt = BFGS(slab, trajectory='slab.traj', logfile='slab.log')

opt.run(fmax=0.05)

write('slab.xyz', slab)
```

Show hidden output

STEP 2. β-MnO2-Pt + OH surface

Here we use the previously optimized surface and ASE add_adsorbate function. We first locate the platinum atom, save it's position number, and finally add the adsorbate ontop of the platinum atom. Identically to before we optimize the surface + OH using TBLite.

```
###############
##-Adding OH-##
###############

slab_oh = read('slab.xyz')

oh = molecule('OH')
oh.rotate(90, 'y')

##-Adsorbent height
h = 2

##-Pt-OH
Pt_idx = [i for i, a in enumerate(slab_oh) if a.symbol == 'Pt'][0]
```

```python
Pt_pos = slab_oh[Pt_idx].position[:2]

add_adsorbate(
    slab_oh,
    adsorbate=oh,
    height=h,
    position=Pt_pos,
)
```

```python
##########################################
##-optimization of MnO2-Pt + OH(1,1,0)-##
##########################################

slab_oh.calc = TBLite(
    method='GFN1-xTB',
    charge=0,
    multiplicity=1,
    spin_polarization=0,
    max_iterations=350,
)

opt = BFGS(slab_oh, trajectory='slab_oh.traj', logfile='slab_oh.log')

opt.run(fmax=0.05)

write('slab_oh.xyz', slab_oh)
```

Show hidden output

```python
view(slab_oh, viewer='x3d')
```

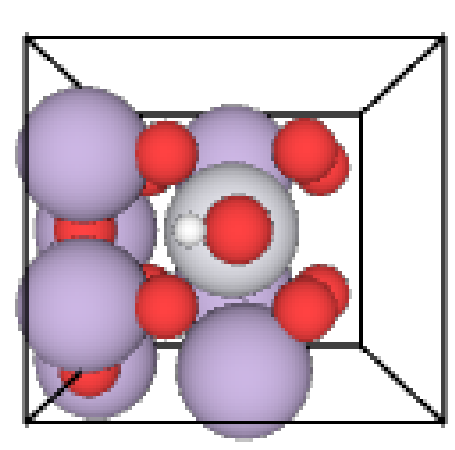

STEP 3. Ciderpress calculation

Now we use the previously optimized β-MnO2-Pt + OH system to run a single-point calculation using the Ciderpress XC functionals. For the sake of time, we set the magnetic moments to 0 for all atoms. We also lowered the convergence criterium with the same intention.

```
#################################
##-ciderpress for OH adsorbate-##
#################################

##-Optimized structure .xyz
slab_oh_opt = read('slab_oh.xyz')

##-setting magmoms for each atom as 0 for the example with Ciderpress
slab_oh_opt.set_initial_magnetic_moments([0.0] * len(slab_oh_opt))

#lab_oh_opt.pbc = (True, True, True)

mlfunc = 'CIDER23X_SL_GGA.yaml'

xc = get_cider_functional(
    mlfunc,
    xmix=0.25,
    qmax=300,
    lambd=1.8,
    pasdw_ovlp_fit=True,
    use_paw=False,
)

slab_oh_opt.calc = CiderGPAW(
    h=0.20,
    xc=xc,
    mode='lcao',
    basis='dzp',
    txt='-',
    occupations={'name': 'fermi-dirac', 'width': 0.10},
    kpts={'size': (2, 2, 1)},
    convergence={'energy': 1e-4, 'density': 1e-3, 'eigenstates': 1e-6},
    mixer=MixerFull(beta=0.05, nmaxold=5, weight=50),
    spinpol=False,
)

etot = slab_oh_opt.get_potential_energy()
```

Show hidden output

```python
view(slab_oh_opt, viewer='x3d')
```

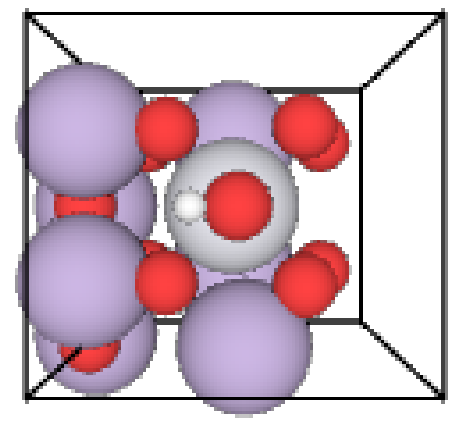

Conclusive remarks

In this tutorial, we started with the crystal structure of β-MnO_2 and transformed it into a catalytic surface using ASE. After creating the (110) slab, we introduced a Pt atom to represent an active site and relaxed the structure using the lightweight TBLite method. Next, an OH adsorbate was placed on the Pt site and the system was optimized again to obtain a stable surface–adsorbate configuration. With the structure prepared, we performed a single-point calculation using a CIDER machine-learned exchange–correlation functional through ciderpress, demonstrating how ML-enhanced DFT can be incorporated into a practical workflow.

After completing this tutorial, you will be able to:

- Perform geometry optimizations with TBLite to efficiently relax both the clean surface and the surface–adsorbate system.

Tutorial 2. Gaussian process structure optimization

Goal of the notebook

The goal of this notebook is to provide step-by-step instructions on how to use GPmin methods to directly enhance DFT simulations.

Description of the method

GPmin attempts to reduce the number of DFT evaluations required during geometry optimization. It does this by learning the relations between atomic configurations, energies and forces from a training set. Using this information it constructs a surrogate potential energy surface, which approximates the true energy surface, but is much cheaper to minimize. The surrogate potential energy surface predicted structure is evaluated with the calculator and added to the local training set. This process is repeated until convergence.

GPmin gitlab: https://gitlab.com/gpatom/ase-gpatom

GPmin article: Garijo del Río, E.; Mortensen, J. J.; Jacobsen, K. W. Local Bayesian Optimizer for Atomic Structures. Phys. Rev. B 2019, 100 (10), 104103. https://doi.org/10.1103/PhysRevB.100.104103.

Outline of the tutorial

STEP 1. PtC3N4-OH creation and mock relaxation

STEP 2. Lattice optimisation with GP method

Installation of packages

Firstly we install the necessary packages

```
##################
##-Installation-##
##################

##-Installing packages
%%capture
!pip install ase scikit-optimize tblite==0.4.0
```

Next we import the necessary packages:

1. ASE https://ase-lib.org/
2. scikit-optimize https://scikit-optimize.github.io/stable/
3. TBLite https://tblite.readthedocs.io

```
##########################
##-Imports for building-##
##########################

##-Imports for ASE
from ase import Atoms
from ase.constraints import FixAtoms
from ase.data.pubchem import pubchem_atoms_search
from ase.optimize import GPMin
from ase.io import read, write
from ase.visualize import view

##-Imports for scikit-optimize
from skopt import gp_minimize
from skopt.plots import plot_convergence, plot_gaussian_process
from skopt.space import Real

##-Import for TBLite
from tblite.ase import TBLite

##-General imports
from numpy import diag
import numpy as np
```

STEP 1. PtC3N4-OH creation and mock relaxation

We first load a template structure, which we modify to make the final structure. The modifications with the explanations can be seen in the code. Next we run a optimise the structure. Since this would take a fairly long amount of time, we "cheat" by importing a nearly optimised structure.

```
#########################################
##-Building initial catalyst structure-##
#########################################

##-Load template structure
PtC3N4 = pubchem_atoms_search(smiles="C1(=NC2=NC(=N)N=C3N2C(=NC(=N3)N)N1)N")

##-Rotate so the 7th and 8th atoms' vector lies along +x (rotate about atom 8 to avoid drift)
v = PtC3N4[7].position[:2] - PtC3N4[8].position[:2]
angle_deg = np.degrees(np.arctan2(v[1], v[0]))
PtC3N4.rotate(-angle_deg, 'z', center=PtC3N4[8].position)

##-Translate so the 8th atom is at the origin
PtC3N4.translate(-PtC3N4[8].position)

##-Set the unit cell
a_PtC3N4 = PtC3N4.get_distance(7, 8)
PtC3N4.set_cell([[a_PtC3N4, 0, 0], [a_PtC3N4/2, a_PtC3N4*(3**0.5)/2, 0], [0, 0, 15]], scale_atoms=False)
PtC3N4.set_pbc([True, True, False])

##-Delete atoms
del PtC3N4[9]
del PtC3N4[7]
del PtC3N4[[atom.index for atom in PtC3N4 if atom.symbol == 'H']]

##-Center at Pt
PtC3N4 += Atoms('Pt', positions=[[a_PtC3N4, a_PtC3N4*(3**0.5)/3, 0]])

##-Move the molecule so that Pt is at [a_PtC3N4/2,(a_PtC3N4/2+a_PtC3N4*(3**0.5)/2)/2,4]
shift = PtC3N4.cell[:2,:2].sum(axis=0) / 2 - PtC3N4[-1].position[:2]
PtC3N4.translate([shift[0], shift[1], 4])

write('PtC3N4.xyz',PtC3N4)

view(PtC3N4, viewer='x3d')
```

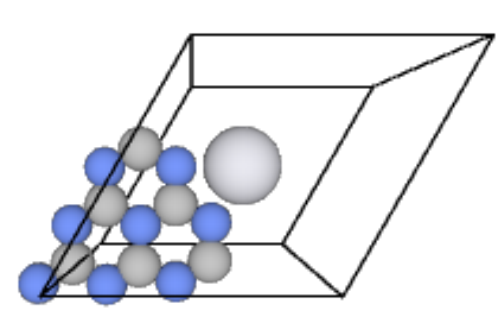

```
#######################################
##-Adding adsorbate to the catalyst-##
#######################################

PtC3N4_OH = PtC3N4 + Atoms('O', positions=[PtC3N4[-1].position + np.array([0,0,2.2])])
PtC3N4_OH += Atoms('H', positions=[PtC3N4_OH[-1].position + np.array([0,0.7,0.7])])

view(PtC3N4_OH, viewer='x3d')
```

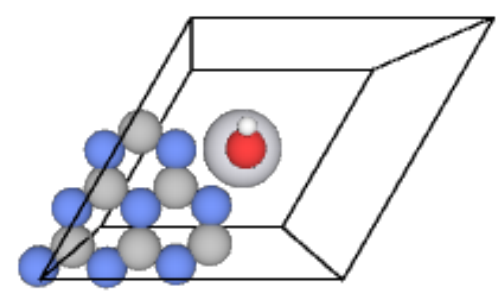

```
##################################################
##-Downloading an almost optimized structure-##
##################################################

%%capture
##-Getting a structure that is almost relaxed since running it from the start is going to take to long for the example.
!wget https://raw.githubusercontent.com/doublelayer/test_models/refs/heads/main/PtC3N4/PtC3N4_OH_lat7_near_relaxed.xyz

view(PtC3N4_OH, viewer='x3d')
```

```
################################
##-Optimizing the structure-##
################################

##-Set calculator
PtC3N4_OH.set_constraint(FixAtoms(indices=[at.index for at in PtC3N4_OH if at.symbol not in ['O', 'H']]))
calculator = TBLite(method="GFN1-xTB")
PtC3N4_OH.calc = calculator

##-Run optimization
dyn = GPMin(PtC3N4_OH, trajectory='PtC3N4_OH_relaxed.traj')
dyn.run(fmax=0.03)

##-Result
view(PtC3N4_OH, viewer='x3d')
write('example_2_gp_min_final.xyz',PtC3N4_OH)
```

Show hidden output

STEP 2. Lattice optimisation with GP method

Here we define a simple lattice calculator based on the Gaussian process method from which scikit learns. This calculator can be adapted towards any problem.

It works by defining a function that creates a structure with a given lattice parameter.The function then calculates the energy and returns it. This way, the entire calculation is encapsulated within a single function that takes the structure as input and returns only the energy. Since scikit learns with pure functions, the varied input arguments and return values must be numerical.

```
##########################
##-Lattice optimization-##
##########################

##-How to use GP_min from scikit to optimise any problem or parameter
##-First example will be a simple problem of lattice parameter optimisation, often this can be done via Stress tensor implem
##-For simplicity we will not be converging the structure at every step.

##-First we start bt defining the function that will be called at every optimisation step.
def lattice_calc(a: float, calculator) -> float:
    ##-As before we use the same the same steps for building the model, such that each opt step start with a fresh model.
    ##-Load the template structure.
    PtC3N4 = pubchem_atoms_search(smiles="C1(=NC2=NC(=N)N=C3N2C(=NC(=N3)N)N1)N")

    ##-Rotate so the 7-8 vector lies along +x (rotate about atom 8 to avoid drift)
    v = PtC3N4[7].position[:2] - PtC3N4[8].position[:2]
    angle_deg = np.degrees(np.arctan2(v[1], v[0]))
    PtC3N4.rotate(-angle_deg, 'z', center=PtC3N4[8].position)
```

```
    ##-Translate so atom 8 is at the origin
    PtC3N4.translate(-PtC3N4[8].position)

    ##-Set the unit cell
    a_PtC3N4 = a #PtC3N4.get_distance(7, 8)
    PtC3N4.set_cell([[a_PtC3N4, 0, 0], [a_PtC3N4/2, a_PtC3N4*(3**0.5)/2, 0], [0, 0, 15]], scale_atoms=False)
    PtC3N4.set_pbc([True, True, False])

    ##-Delete atoms
    del PtC3N4[9]
    del PtC3N4[7]
    del PtC3N4[[atom.index for atom in PtC3N4 if atom.symbol == 'H']]

    ##-Center at Pt
    PtC3N4 += Atoms('Pt', positions=[[a_PtC3N4, a_PtC3N4*(3**0.5)/3, 0]])
    shift = PtC3N4.cell[:2,:2].sum(axis=0) / 2 - PtC3N4[-1].position[:2]
    PtC3N4.translate([shift[0], shift[1], 4])

    ##-Setting the calculation, and changing the txt as to not overwrite it
    PtC3N4.calc = calculator
    PtC3N4.calc.txt = f'PtC3N4_OH_a{a}.txt'

    ##-Calculating the potential energy.
    ##-The step function needs to return a single value to serve as objective value for the optimiser.
    return PtC3N4.get_potential_energy()

calculator = TBLite(method="GFN1-xTB")

##-The gp_min object
gp_min_obj = gp_minimize(
    ##-We here decorate the function with a lambda function, thereby setting the static arguments that does not change durin
    ##-*par_list means the gp_minimize is able to optimize more than one variable at the same time, but in this case 1, the
    func= lambda par_list: lattice_calc(*par_list, calculator=calculator),
    ##-Each parameter to optimise have to be defined as a dimensions with an upper and lower bound
    dimensions= [Real(5, 10)], ##-Guessing the energy minima is between 5 and 10 Å
    n_calls = 10, ##-The number of iterations to run, this gp_min does not appear to have convergen criteria...
    n_initial_points = 3, ##-Do 3 calculations at random points before beginning to estimate the space
    random_state = 42, ##-Sets the random state for reproduction
    verbose= True, ##-For testing
)
```

Show hidden output

```
print(f'minimum was found to be at {gp_min_obj.x} Å with and energy of {gp_min_obj.fun} eV')
plot_convergence(gp_min_obj)
plot_gaussian_process(gp_min_obj)
```

```
minimum was found to be at [7.793299974075221] Å with and energy of -1160.7696240341957 eV
<Axes: title={'center': 'x* = 7.7933, f(x*) = -1160.7696'}, xlabel='x', ylabel='f(x)'>
```

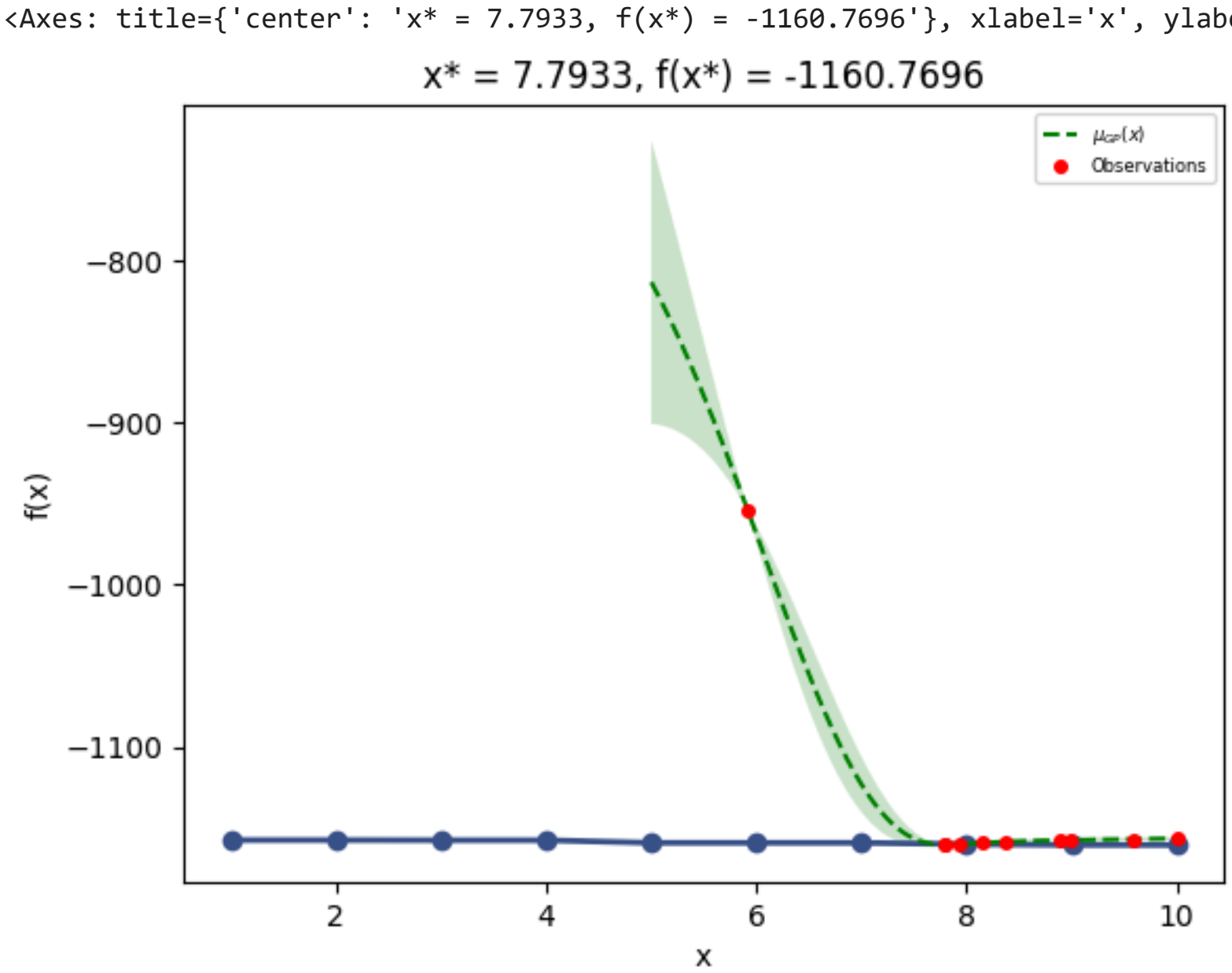

Concluding remarks

In this tutorial we performed a standard geometry optimization of a platinum graphitic carbon nitride catalyst with an OH intermediate on top, but instead of the standard Broyden-Fletcher-Goldfarb-Shannon (BFGS) algortihm with GPmin machine-learning algorithm.

This substitution illustrates how machine-learning methods can be used to enhance DFT simulations by guiding the search toward optimal structures more efficiently.

After completing this tutorial we can:

- Use the GPMin optimmizer to perform geometry optimization within ASE
- Run GPMin-based optimizations
- Understand how Gaussian process optimization can be used to find best values of parameters (example lattice parameters)

Tutorial 3. Bayesian statistics in search for energy extrema

Goal of the notebook

The goal of this notebook is provide step-by-step instructions on how to use the BOSS method to find the adsoprtion site of OH intermediate on a PtN_4-carbon single-atom catalyst.

Description of the method

Bayesian Optimisation Structure Search (BOSS) is a method for exploring atomistic configuration spaces and identifying stable structures using a small number of energy evaluations. The method combines Bayesian optimisation with DFT calculations to model the potential energy surface during the search. A surrogate model, usually a Gaussian process, is updated with energies computed for selected atomic configurations. New configurations are selected to sample unexplored regions of the configuration space and configurations with lowest energy.

BOSS is used for problems where each energy evaluation is expensive, such as density functional theory calculations of adsorption structures or molecular configurations on surfaces. During the search the model learns the energy landscape and identifies global and local minima without grid sampling or manual selection of structures.

BOSS webpage: https://cest-group.gitlab.io/boss/tutorials.html

BOSS github: https://gitlab.com/cest-group/boss

BOSS article: Todorović, M.; Gutmann, M. U.; Corander, J.; Rinke, P. Bayesian Inference of Atomistic Structure in Functional Materials. *npj Computational Materials* **2019**, *5*, 35. https://doi.org/10.1038/s41524-019-0175-2.

Outline of the tutorial

STEP 1. Creating the surface + adsorbate

STEP 2. Finding the asdorption site for OH

The tutorial takes less than 2 minutes to run on 1 CPU in Google Colab.

Installation of packages

First thing first, we need to install the packages.

```
##################
##-Installation-##
##################

!pip install ase aalto-boss tblite==0.4.0
```

Show hidden output

Next we import the necessary packages:

1. ASE https://ase-lib.org/
2. BOSS https://cest-group.gitlab.io/boss
3. TBLite https://tblite.readthedocs.io

```
##############
##-Imports-###
##############

##-Imports for ASE
from ase import Atoms, Atom
from ase.build import fcc111, graphene
from ase.io import read, write
from ase.visualize import view

##-Imports for Boss
from boss.bo.bo_main import BOMain

##-Import for TBLite
from tblite.ase import TBLite

##-General imports
import numpy as np
```

STEP 1. Creating the surface + adsorbate

At the first step, we create the surface using ASE functions and run single point calculations on H_2O, H_2 and OH.

```
##########################
##-Creating the surface-##
##########################
```

```python

# Start with a graphene sheet
PtN4 = graphene(size=(4,4,1))
# Substitute 4 C atoms with N, add Pt and remove 2 C atoms
for i in (0, 3, 8, 11):
    PtN4[i].symbol = "N"
PtN4.append(Atom('Pt', position=(PtN4[1].position+PtN4[10].position)/2))
del PtN4[[10, 1]]
# Change the unit cell, shift Pt to the center
PtN4.cell[1,0] *= -1
PtN4.center(vacuum=5, axis=2)
shift = PtN4.cell[:2,:2].sum(axis=0) / 2 - PtN4[-1].position[:2]
PtN4.translate([shift[0], shift[1], 0.0])
PtN4.wrap()
PtN4.pbc = (True, True, False)
```

```python
####################
##-Defining species-##
######################

##-This sets up species needed for calculations
OH       = Atoms('OH', positions=[[0,0,0], [0,0,0.96]])
water    = Atoms('H2O',positions=[[0.75,0.58,0],[-0.75,0.58,0],[0,0,0]])
hydrogen = Atoms('H2', positions=[[0,0,0],[0.7,0,0]])
```

```python
###############################
##-Single point calculations-##
###############################

PtN4.calc     = TBLite(method="GFN1-xTB")
water.calc    = TBLite(method="GFN1-xTB")
hydrogen.calc = TBLite(method="GFN1-xTB")

slab_energy  = PtN4.get_potential_energy()
water_energy = water.get_potential_energy()
hydro_energy = hydrogen.get_potential_energy()
```

Show hidden output

STEP 2. Finding the preferred asdorption site for OH

At the second step, we define two functions. The first function places our adsorbate on 6 coordinates. The second function represents BOSS taking the coordinates and returning a single scalar. Now, using the defined functions and the BOSS object, we run the calculations to get the predicted global energy minimum.

```
########################
##-Defining functions-##
########################

##-This function will place our adsorbate on given 6 coordinates
def build_system(x,y,z,rx,ry,rz, slab: Atoms, adsorbate: Atoms,) -> Atoms:
    slab = slab.copy()
    adsorbate = adsorbate.copy()
    adsorbate.rotate(rx,'x')
    adsorbate.rotate(ry,'y')
    adsorbate.rotate(rz,'z')
    adsorbate.translate((x,y,z))
    full_system = slab + adsorbate
    return full_system

##-This function represents one step of BOSS taking the 6 dimensional coorc
def calculate_system(x,y,z,rx,ry,rz, slab: Atoms, adsorbate: Atoms, slab_er
    full_system = build_system(x,y,z,rx,ry,rz, slab, adsorbate)
    full_system.calc = TBLite(method="GFN1-xTB", verbosity = 0)
    full_system.calc.txt = f'full_system_{x}-{y}-{z}_{rx}-{ry}-{rz}.txt'
    if isinstance(adsorbate_energy, list) or isinstance(adsorbate_energy, t
    return full_system.get_potential_energy() - slab_energy - adsorbate_ene
```

```
##########################
##-Optimizing structure-##
##########################

BO_obj = BOMain(
    f= lambda args: calculate_system(args[0,0],args[0,1],args[0,2],args[0,3
                                     PtN4, OH,
                                     slab_energy=slab_energy,
                                     adsorbate_energy=(water_energy, -0.5*h
    bounds=np.array([[3/7*(PtN4.cell[0,0] + PtN4.cell[1,0]), 4/7*(PtN4.cell
                     [ 2/5*PtN4.cell[1,1], 3/5*PtN4.cell[1,1]],
                     [2/3*PtN4.cell[2,2], 3/4*PtN4.cell[2,2]],
                     [-30, 30],
                     [-30, 30],
                     [  0, 60],
                      ]
                     ),
    kernel='rbf',
    initpts=5,
    iterpts=16
)

results = BO_obj.run()

print('Predicted global min: ', results.select('mu_glmin', -1))
print(results.select('x_glmin', -1))
```

Show hidden output

```
PtN4_opt = build_system(*results.select('x_glmin', -1), slab = PtN4, adsort
PtN4_opt.wrap()
view(PtN4_opt, viewer='x3d')
```

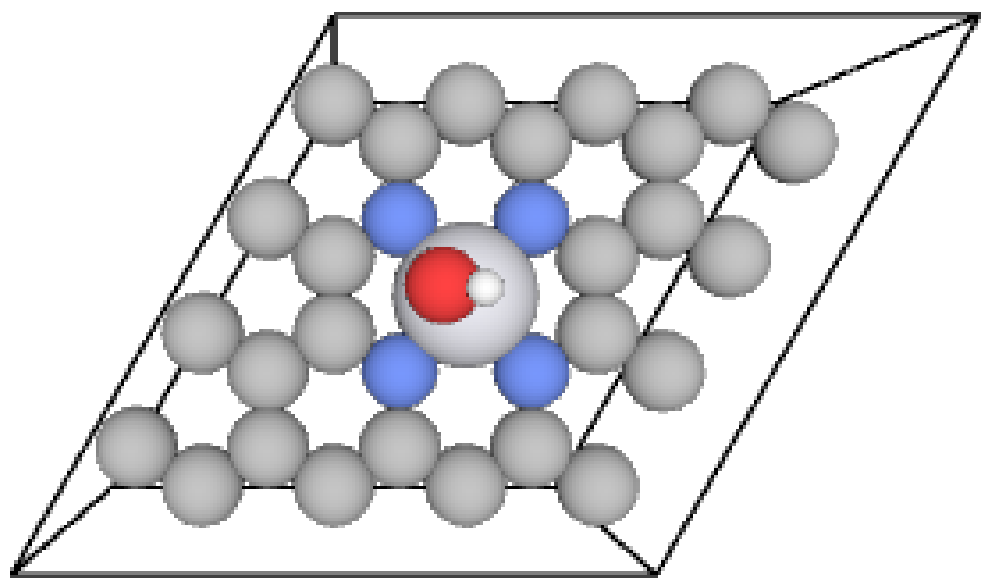

Concluding remarks

In this tutorial we applied BOSS to study OH adsorption on a PtN_4–carbon single-site catalyst. We searched for the configuration with the lowest adsorption energy. This search identified that OH prefers to adsorb ontop Pt atom. This is the adsorption site a chemist would expect to be most reactive.

This example shows how BOSS can screen thought adsorption configurations to learning the energy surface and reveal the lowest energy configurations.

After completing this tutorial we can:

- Set up and run a BOSS search for an adsorption problem.
- Define the degrees of freedom that describe the adsorbate configuration.
- Build and update a Gaussian process model of the adsorption energy surface during the search.
- Use the model to guide the search toward the lowest-energy adsorption configuration.

Tutorial 4. Bayesian statistics in configuration search

Goal of the notebook

The goal of this example is to demonstrate the use of the BEACON as implemented in GPAtom and ASE to a 13-atom Ag-Pd-Pt-Ru nanoparticle.

Description of the method

The BEACON method applies Bayesian optimization to efficiently search complex configurational spaces in multimetallic systems. Atomic structures are represented using global structural fingerprints that encode both composition and geometry, and their energies are modeled with a Gaussian process surrogate model. During the search, new candidate configurations are generated and evaluated, while the surrogate model guides the exploration toward low-energy structures. This approach enables efficient identification of stable atomic arrangements in alloy nanoparticles, where compositional ordering and atomic positions strongly influence catalytic properties.

BEACON gitlab: https://gitlab.com/gpatom/ase-gpatom

BEACON article: Kaappa, S.; del Río, E. G.; Jacobsen, K. W. Global Optimization of Atomic Structures with Gradient-Enhanced Gaussian Process Regression. Phys. Rev. B 2021, 103 (17), 174114. https://doi.org/10.1103/PhysRevB.103.174114 .

Outline of the tutorial

STEP 1. Nanoparticle search

STEP 2. Place OH on the lowest energy nanoparticle

STEP 3. Best adsorption location search

Installation of packages

First thing first, we need to install the packages and import the neccessary functions.

```
##################
##-Installation-##
##################

%%capture
!pip install ase tblite==0.4.0 ase-gpatom
```

Next we import the necessary packages:

ASE https://ase-lib.org/

TBLite https://github.com/tblite

GPAtom https://gitlab.com/gpatom

```
#############
##-Imports-##
#############

##-ASE
from ase import Atoms, Atom
from ase.io import read, write
from ase.visualize import view
from ase.data import covalent_radii, atomic_numbers
from ase.constraints import FixAtoms, FixBondLengths

##-TBLite
from tblite.ase import TBLite

##-GPAtom
from gpatom.gpfp.prior import RepulsivePotential, CalculatorPrior
from gpatom.beacon.str_gen import  RandomBox, AtomsRelaxer, RandomMolecule, RandomStructure
from gpatom.gpfp.gp import GaussianProcess
from gpatom.gpfp.fingerprint import FingerPrint, FPUpdater
from gpatom.gpfp.avishart_hpfitter import HpFitterConstantRatioParallel
```

```
from gpatom.gpfp.atoms_gp_interface import LCBModel
from gpatom.beacon.beacon import  (BEACON, SurrogateOptimizer,InitatomsGenerator, Checker)

##-Other
import numpy as np
```

STEP 1. Nanoparticle Search

BEACON generates random atomic arrangements of the alloy and evaluates their energies using TBLite calculator. This energy is inturn used by a Gaussian-process model surrogate which guides the search toward promising structures, essentially locating the low-energy geometries.

```
########################
##-Creating the alloy-##
########################

##-Creating the alloy
elements = Atoms(['Pt']*3 + ['Pd']*2 + ['Ag']*3 + ['Ru']*5,  cell = [15,15,15], pbc=False)
elements.calc = TBLite(method="GFN1-xTB", verbosity = 0)
```

```
#################################
##-Structure generation setup-##
#################################

##-First we define a repulsive potential to make sure that atoms arent placed ontop  of each other, this is a simple lennar
potential=RepulsivePotential(prefactor=10, rc=0.9)

##-Then a relaxtion algoritm using the potential as calculator, This is an interface that call BFGS, it might be replaceabl
relaxer=AtomsRelaxer(calculator=potential, with_unit_cell=False)

##-The random generator, we dont have multple ranks so no need to account for it in this instance
rng = np.random.RandomState(42)

system_gen = RandomBox(elements, box=[(5, 10)]*3, rng=rng, relaxer=relaxer)
initatomsgen=InitatomsGenerator(sgen=system_gen, rgen=system_gen)

##-Now we have all the code for generating structures
```

```
###########################
##-Surrogate model setup-##
###########################

##-Sets and interfaces the calculator used in fingerprinting
prior_calculator = potential ##-TBLite(method="GFN1-xTB", verbosity = 0)
prior=CalculatorPrior(potential, constant=0)

gp_args = dict(prior=prior,
               hp={'scale': 1000, 'weight': 100,
                   'ratio': 0.001, 'noisefactor': 1},
               use_forces=True)

gp=GaussianProcess(**gp_args)


##-Next we set the size of a Atom fingerprint to  that of the largest atom
r_atom = max(covalent_radii[atomic_numbers['Pt']],
             covalent_radii[atomic_numbers['Pd']],
             covalent_radii[atomic_numbers['Ag']],
             covalent_radii[atomic_numbers['Ru']])
fp_args = {'r_cutoff': r_atom*5, 'a_cutoff': r_atom*3, 'aweight': 1}
fp=FingerPrint(fp_args=fp_args, calc_strain=False)

##-Optimiser for parameters
hp_optimizer = HpFitterConstantRatioParallel()

##-Fingerprint updater
fp_updater=FPUpdater(factor=1/3)

##-Finally everything is put together in the ML model
model=LCBModel(gp=gp, fp=fp, hp_optimizer=hp_optimizer, fp_updater=fp_updater, kappa=2)
```

```
###################
##-BEACON search-##
###################

surropt=SurrogateOptimizer(fmax=0.05, relax_steps=100,
                           with_unit_cell=False)

##-The checker check structure likeness
checker=Checker(dist_limit=0.5, rlimit=0.4)

calculator = TBLite(method="GFN1-xTB", verbosity = 0)

go = BEACON(calculator,
            model,
            initatomsgen,
            surropt=surropt,
            checker=checker,
            ninit=2, ##-How many training points we want in initial set
            ndft=10, ##-How many TBlite calculations are done in total
            nsur=3, ##-How many surrogate optimizations are done per cycle
            write_surropt=True, ##-Save surrogate relaxation end structures
            write_surropt_trajs=False) ##-Dont save trajectory files for all surrogate relaxations
##-Run:
go.run()
```

Show hidden output

STEP 2. Placing adsorbate

After the search completes, we choose the lowest-energy nanoparticle structure identified by BEACON. Onto that nanoparticle, we generate an OH molecule, positioned near the nanoparticle while avoiding overlapping. Nanoparticles atom positions are fixed.

```
####################
##-Best candidate-##
####################

best_candidate = go.get_candidate()[0]
write('example_4_BEACON_best_result.xyz', best_candidate)
```

```
#################
##-Definitions-##
#################

def sph2cart(r, theta, phi):
    """Convert spherical to Cartesian coordinates."""
    x = r * np.sin(theta) * np.cos(phi)
    y = r * np.sin(theta) * np.sin(phi)
    z = r * np.cos(theta)
    return x, y, z

def cart2sph(x, y, z):
    """Convert Cartesian to spherical coordinates."""
    r = np.sqrt(x**2 + y**2 + z**2)
    theta = np.arccos(z / r)            ##-Polar angle
    phi = np.arctan2(y, x)              ##-azimuth
    return r, theta, phi



def cell_center_cartesian(atoms):
    """Return the Cartesian coordinates of the geometric center of the unit cell."""
    cell = atoms.get_cell()          ##-shape (3,3), cell vectors as rows
    frac_center = np.array([0.5, 0.5, 0.5])
    ##-fractional -> cartesian
    cart_center = frac_center.dot(cell)
    return cart_center


class OHGenerator:
    def __init__(self, rng, relaxer=None):
        self.rng=rng

    def get(self):

```

```
        atoms = Atoms('OH', positions=[[0,0,0], [0,0,0.96]])
        atoms.constraints.append(FixBondLengths([[0,1]]))

        for axis in ['x', 'y', 'z']:
            angle = self.rng.uniform(0, 360)
            atoms.rotate(angle, axis, rotate_cell=False)
        return atoms


class MoleculeAroundCenter():
    """
    Generator for randomly placing molecules a structure at the center of the box.
    the generator doesn't change the molecule itself

    this functions is a modified copy of MoleculeOnSubstrate

    Examples:
    >>> sgen=MoleculeOnSubstrate(molecule_generator, adsorbate, dx=2)
    >>> new_atoms=sgen.get()
    """
    def __init__(self, molecule_generator, substrate,  dr=3,
                 relaxer=None, **kwargs):

        """
        Parameters
        ----------
        molecule_generator: BEACON structure generator object
            A structure generator with a method molecule=molecule_generator.get()
            taking no inputs and outputting a molecule subject to custom
            generation logic, including setting of molecular constraints.
            MoleculeOnSubstrate knows how to handle molecules constrained by
            FixAtoms and FixBondLengths
            Required.

        substrate : ase.Atoms
            The substrate structure on which the adsorbate will be placed.
            Required.

        dr : float, optional
            Displacement interval in which the molecule can be placed from
            the center.
            Default is 3 Angstrom.

        relaxer : Relaxer object, optional
            Relaxation procedure applied after structure generation.
            Default is None. (No relaxation)

        **kwargs
            Additional keyword arguments passed to RandomStructure.
            May include:

            rng : random number generator, optional
                Random number generator for reproducibility.
                Default is `np.random` (not reproducible).
        """


        RandomStructure.__init__(self, substrate, **kwargs)
        self.molecule_generator=molecule_generator
        self.substrate=substrate
        self.dr=dr
        self.relaxer=relaxer

    def get(self):

        mol=self.molecule_generator.get()

        substrate=self.substrate.copy()
        mol.cell=substrate.cell.copy()

        ##-get the center of the box in both cartesian and spherical coordinates
        cell=substrate.get_cell()
        cell_center=cell_center_cartesian(mol)
        cell_center_sph=cart2sph(*cell_center)

        ##-this will rely on the mol com not being origo for getting the oriantation of the molecule
        mol_center = mol.get_center_of_mass()
```

```
        mol_center_sph = cart2sph(*mol_center)
        mol_center_uv = mol_center/np.linalg.norm(mol_center)

        radia_shift = self.rng.random() * self.dr + 1.05*self.get_minimum_distance(mol_center_uv, np.array(cell_center))
        random_shift = sph2cart(radia_shift,mol_center_sph[1],mol_center_sph[2])

        ##-moving the molecule to the center of the substrate cell
        mol.translate(cell_center)
        ##-moving the molecule in accordance with the random shift
        mol.translate(random_shift)

        atoms=substrate+mol
        atoms.pbc=[True, True, True]

        offset = len(substrate)

        constraints = []
        for c in substrate.constraints:
            constraints.append(c)

        for c in mol.constraints:
            if isinstance(c, FixAtoms):
                new_indices = [i + offset for i in c.index]
                constraints.append(FixAtoms(indices=new_indices))
            elif isinstance(c, FixBondLengths):
                new_bonds = [[i + offset, j + offset] for (i, j) in c.pairs]
                constraints.append(FixBondLengths(new_bonds))

        atoms.set_constraint(constraints)

        if self.relaxer is not None:
            atoms=self.relaxer.run(atoms)

        return atoms


    def get_minimum_distance(self, O_center_unit_vector, center) -> float:
        min_distance = 0
        for at in self.substrate:
            distance_from_at_O_vector = (center - at.position) - np.dot(np.dot((center - at.position), O_center_unit_vectc
            collision_size = covalent_radii[atomic_numbers['O']] + covalent_radii[atomic_numbers[at.symbol]]
            if np.linalg.norm(distance_from_at_O_vector) < collision_size:
                collision_distance_from_center = np.linalg.norm(np.dot(np.dot((center - at.position), O_center_unit_vector
                if collision_distance_from_center > min_distance: min_distance = collision_distance_from_center
            else: continue
        return min_distance


```

```
########################
##-OH placement setup-##
########################

##-Now lets ud try to add OH
##-First we will fix all the atoms we dont want the generation to move about
nano_catalyst = best_candidate.copy()
nano_catalyst.set_constraint(FixAtoms(indices=[at.index for at in nano_catalyst]))

##-Next add the OH to the system
##-OH = Atoms('OH', positions=[[0,0,0], [0,0,0.96]])
##-Nano_catalyst += OH

##-First we define a repulsive potential to make sure that atoms arent placed ontop  of each other, this is a simple lennar
potential=RepulsivePotential(prefactor=10, rc=0.9)

##-Then a relaxtion algoritm using the potential as calculator, This is an interface that call BFGS, it might be replaceabl
relaxer=AtomsRelaxer(calculator=potential, with_unit_cell=False)

##-The random generator, we dont have multple ranks so no need to account for it in this instance
rng = np.random.RandomState(42)

system_gen = MoleculeAroundCenter(OHGenerator(rng=rng), nano_catalyst, dr=5, rng=rng, relaxer=relaxer)
initatomsgen=InitatomsGenerator(sgen=system_gen, rgen=system_gen)

##-Now we have all the code for generating structures
```

```
print(nano_catalyst)
view(nano_catalyst, viewer='x3d')
```

```
Atoms(symbols='Pt3Pd2Ag3Ru5', pbc=False, cell=[15.0, 15.0, 15.0], constraint=FixAtoms(indices=[0, 1, 2, 3, 4, 5, 6, 7, 8, 9, 10,
```

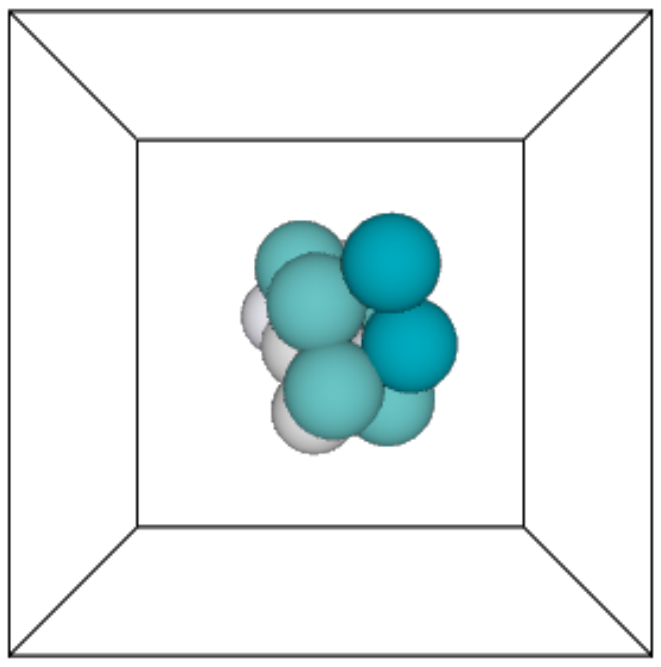

STEP 3. Adsorbate

We use BEACON again to explore different OH placements around the nanoparticle. The model finds the most stable OH adsorption configuration. When the lowest energy nanoparticle is found, we save the best result.

```
##############################
##-Adsorption search setup-##
##############################

##-Set up a new model same as before
##-Sets and interfaces the calculator used in fingerprinting
prior_calculator = potential #TBLite(method="GFN1-xTB", verbosity = 0)
prior=CalculatorPrior(potential, constant=0)

gp_args = dict(prior=prior,
               hp={'scale': 1000, 'weight': 100,
                   'ratio': 0.001, 'noisefactor': 1},
               use_forces=True)

gp=GaussianProcess(**gp_args)


##-Next we set the size of a Atom fingerprint to  that of the largest atom
##-We will keep this as is since the size of the metals still matter and are so much larger than the adsorbate radii we are
r_atom = max(covalent_radii[atomic_numbers['Pt']],
             covalent_radii[atomic_numbers['Pd']],
             covalent_radii[atomic_numbers['Ag']],
             covalent_radii[atomic_numbers['Ru']])
fp_args = {'r_cutoff': r_atom*5, 'a_cutoff': r_atom*3, 'aweight': 1}
fp=FingerPrint(fp_args=fp_args, calc_strain=False)

##-Optimiser for parameters
hp_optimizer = HpFitterConstantRatioParallel()

##-Fingerprint updater
fp_updater=FPUpdater(factor=1/3)

##-Finally everything is put together in the ML model
model=LCBModel(gp=gp, fp=fp, hp_optimizer=hp_optimizer, fp_updater=fp_updater, kappa=2)

```

```
###################
##-BEACON search-##
###################

##-Set up a new beacon same as before.
surropt=SurrogateOptimizer(fmax=0.05, relax_steps=100,
                           with_unit_cell=False)

##-The checker check structure likeness
checker=Checker(dist_limit=0.5, rlimit=0.4)


```

```
calculator = TBLite(method="GFN1-xTB", verbosity = 0, max_iterations = 4000)

go = BEACON(calculator,
            model,
            initatomsgen,
            surropt=surropt,
            checker=checker,
            ninit=2, ##-How many training points we want in initial set
            ndft=10, ##-How many TBlite calculations are done in total
            nsur=3, ##-How many surrogate optimizations are done per cycle
            write_surropt=True, ##-Save surrogate relaxation end structures
            write_surropt_trajs=False) ##-Dont save trajectory files for all surrogate relaxations
##-Run:
go.run()
```

Show hidden output

```
#######################
##-Best OH candidate-##
#######################

best_OH_candidate = go.get_candidate()[0]
write('example_4_BEACON_best_OH_result.xyz', best_OH_candidate)
print(best_OH_candidate)
view(best_OH_candidate, viewer='x3d')
```

```
Atoms(symbols='Pt3Pd2Ag3Ru5OH', pbc=True, cell=[15.0, 15.0, 15.0], constraint=[FixAtoms(indices=[0, 1, 2, 3, 4, 5, 6, 7, 8, 9, 1
```

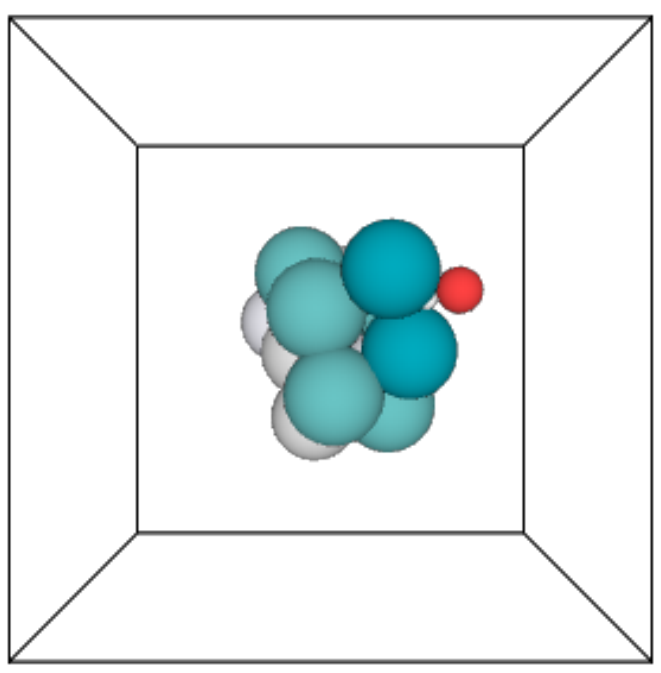

Concluding remarks

In this tutorial, we explored how Bayesian optimization can be used to search complex atomic configurations in multimetallic nanoparticles. We began by generating random structures of a 13-atom $Ag_3Pd_2Pt_3Ru_5$ nanoparticle and used BEACON together with TBLite to identify the lowest-energy arrangement of atoms. Once the most stable nanoparticle configuration was found, an OH molecule was introduced and the search was repeated to determine the most favorable adsorption configuration.

This workflow illustrates how Bayesian optimization and Gaussian-process models can efficiently explore both atomic ordering and adsorption geometries, enabling the identification of stable catalyst structures in complex alloy systems.

After completing this tutorial we can:

- Set up and implement BEACON algorithm
- Generate and relax random alloy nanoparticle structures
- Identify the most stable atomic arrangements
- Perform adsorption site searches

Tutorial 5. ML interatomic potentials for simulations of catalytic surfaces

IMPORTANT

In order to run this notebook you need to request access to the UMA repository on https://huggingface.co/facebook/UMA.

Goal of the notebook

The goal of this example is to demonstrate how it is possible to model a surface with an adsorbate and optimize the geometry using machine-learning potential (UMA)

Description of the method

UMA is a machine-learning interatomic potential trained on large datasets of DFT calculations for catalytic systems. It predicts energies and forces for atomic structures, allowing geometry optimization similar to DFT but at significantly lower computational cost.

UMA github: https://github.com/facebookresearch/fairchem

UMA webpage: https://huggingface.co/facebook/UMA

UMA article: Wood, B. M.; Dzamba, M.; Fu, X.; Gao, M.; Shuaibi, M.; Barroso-Luque, L.; Abdelmaqsoud, K.; Gharakhanyan, V.; Kitchin, J. R.; Levine, D. S.; Michel, K.; Sriram, A.; Cohen, T.; Das, A.; Rizvi, A.; Sahoo, S. J.; Ulissi, Z. W.; Zitnick, C. L.UMA: A Family of Universal Models for Atoms. arXiv 2026, arXiv:2506.23971. https://arxiv.org/abs/2506.23971.

Outline of tutorial

STEP 0. Login to Hugging Face

STEP 1. Build a Pt(111) surface with OH adsorbates.

STEP 2. Load the UMA model and run the geometry optimization.

Installation of packages

First thing first, we need to install the packages and import the neccessary functions.

```
#%%capture
!pip install fairchem-core --upgrade ase
```

Show hidden output

Next we import the necessary packages:

ASE https://ase-lib.org/

fairchem https://fair-chem.github.io/

```
##-ASE
from ase.io import read, write
from ase.constraints import FixAtoms
from ase.optimize import BFGS
from ase import Atoms
from ase.visualize import view

##-Fairchem
from fairchem.core import pretrained_mlip, FAIRChemCalculator

##-General
import os
import torch
import numpy as np
```

STEP 1. Build a Pt(111) surface with OH adsorbates

Now we load the UMA model using get_predict_unit function and selecting the model we want. Next we get the structure of the slab and define it as an atoms object. For defining the atoms object we use trajectory[-1] which gives the last configuration of the optimization process. Lastly we make a directory and set it as the output for trajectory files.

Load the UMA model from this link and upload it to the same folder where this tutorial is placed.

```
%%capture
##-Load UMA model
model_path="/content/uma-s-1p1.pt"

##-Downloading the structure
```

```
!wget https://raw.githubusercontent.com/doublelayer/test_models/main/Pt-w/P

##-Defining atoms object
structure_path = 'Pt111OH_3x4x1.xyz'
trajectory = read(structure_path, index=':')
atoms = trajectory[-1]

##-Make a directory for data, and set the trajectory file output to that di
os.makedirs("data", exist_ok=True)
output_traj_path = "data/Pt_uma_relax1.traj"
```

STEP 2. Load the UMA model and run the geometry optimization

Now we can costumize the calculation object. We chose to set the PBC of the system as periodic in all directions. Next we have to attatch the previously loaded UMA as a calculator to the atoms object. Before choosing the optimizer and running the calculations, we fix the surface atoms inorder to speed up the calculations. Finally we set the optimizer and run the optimization.

```
##-Setting PBC
atoms.pbc = [True, True, True]  # or [False, False, False]

##-Creating ASE calculator
calc = FAIRChemCalculator.from_model_checkpoint(
    model_path,
    task_name="oc20",
    device='cpu',
)

##-Attatching the calculator to atoms object
atoms.calc = calc
##-Adding constraints to atoms
cons = FixAtoms(indices=[atom.index for atom in atoms if atom.tag == 0])
atoms.set_constraint(cons)

##-Run the optimization using BFGS
dyn = BFGS(atoms, trajectory=output_traj_path)
dyn.run(fmax=0.1, steps=100)
```

Show hidden output

```
view(atoms, viewer='x3d')
```

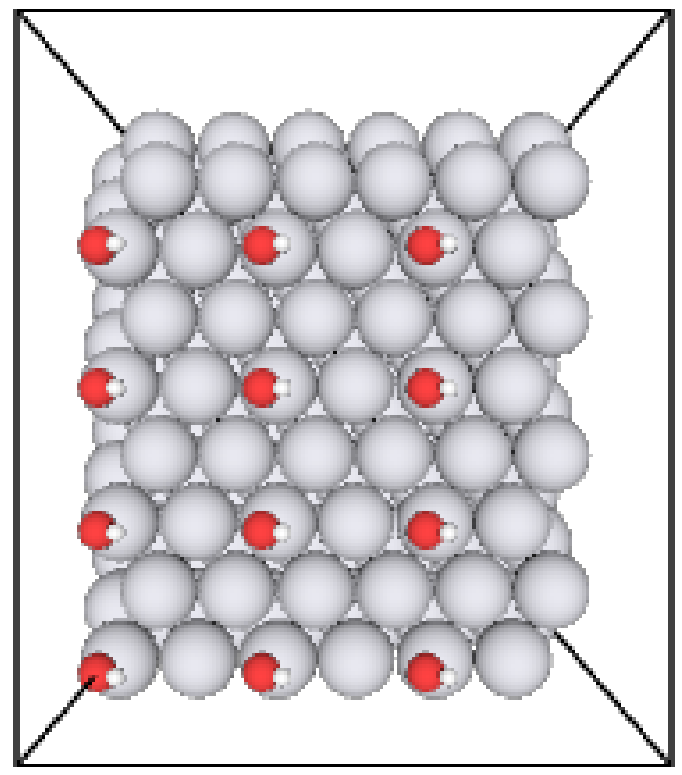

Concluding remarks

In this tutorial we used UMA to model a Pt(111) surface with OH groups. A pretrained model was used to calculate forces and energies to optimize the geometry of Pt(111) with OH adsorbates.

This tutorial demonstrates that the use of machine-learning interatomic potentials of UMA can greatly reduce the optimization time compared to the usual DFT computational time.

After completing this tutorial we can:

- Loadthe UMA machine-learning interatomic potentials
- Run an optimization with UMA as the calculator and BFGS as the optimizer

Tutorial 6. ML force fields to simulate a realistic Pt–OH–H2O interface

The goal of the notebook

This notebook demonstrates how a realistic catalytic interface containing a metal surface, adsorbate, and solvent molecules can be modeled and relaxed using the SpookyNet machine-learning force field.

Description of the method

SpookyNet is a machine-learning force field trained on DFT data that predicts energies and forces while accounting for electronic effects such as polarization and charge transfer. It enables efficient simulations of catalytic interfaces that are difficult to treat with classical force fields.

SpookyNet github: https://github.com/OUnke/SpookyNet

SpookyNet article: Unke, O. T.; Chmiela, S.; Gastegger, M.; Sauceda, H. E.; Müller, K.-R.; Tkatchenko, A. SpookyNet: Learning Force Fields with Electronic Degrees of Freedom and Nonlocal Effects. Nat. Commun. 2021, 12, 7273. https://doi.org/10.1038/s41467-021-27504-0

Outline of the workflow

STEP 1. Build a Pt(111) surface with an OH adsorbate and water molecules.

STEP 2. Run geometry optimization using Spookynet.

Installation of packages

First thing first, we need to install the packages and import the neccessary functions. We need ASE,torch and Spookynet

```
%%capture
!git clone https://github.com/OUnke/SpookyNet.git
%cd SpookyNet
!pip install .
!pip install torch ase
```

Next we import the necessary packages:

ASE https://ase-lib.org/

SpookyNet: https://github.com/OUnke/SpookyNet

Torch: https://github.com/pytorch/pytorch

```
##-ASE
from ase import Atoms, Atom
from ase.constraints import FixAtoms
from ase.io import read, write, Trajectory
from ase.md import MDLogger
from ase.md.langevin import Langevin
from ase.units import fs
from ase.visualize import view
from ase.optimize import BFGS

##-General
import torch
import os

##-matplotlib
import matplotlib.pyplot as plt

##-Spookynet
from spookynet.spookynet_calculator import SpookyNetCalculator
```

STEP 1. Preparing the Pt–OH–H_2O interface

In this step we load a prepared Pt(111)–OH–H_2O structure and create an ASE atoms object. The Pt atoms representing the metal slab are constrained to mimic a bulk surface, while the adsorbate and water molecules remain free to relax.

```
%%capture

##-Downloading the Pt-OH-H2O structure
!wget https://github.com/doublelayer/test_models/raw/refs/heads/main/Pt-w/Pt-OH-H2O.traj
```

```
##-Creating the atoms object
atoms = read(f'Pt-OH-H2O.traj')
view(atoms, viewer='x3d')
```

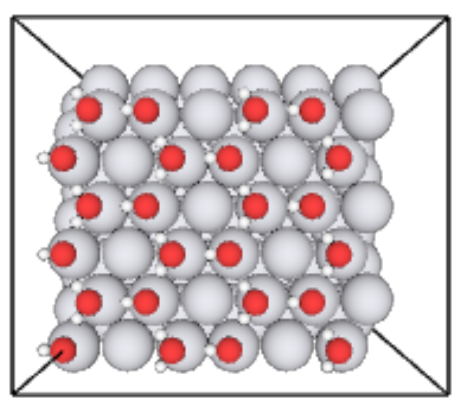

```
##-Adding tag = 1 for each Pt atom
for atom in atoms:
    if atom.symbol == 'Pt':
        atom.tag = 1

##-Constraining atoms with tag = 1
indices = [atom.index for atom in atoms if atom.tag == 1]
atoms.set_constraint(FixAtoms(indices))
```

STEP 2. Geometry optimization with Spookynet

The pretrained SpookyNet model is loaded and assigned as the calculator. It predicts energies and forces for the atomic configuration, replacing traditional DFT calculations in the optimization workflow. Using the predicted forces, a BFGS optimizer iteratively updates atomic positions until the forces fall below the convergence threshold, yielding a relaxed interface structure.

```
atoms.center(vacuum=13.0, axis=2)
atoms.set_pbc(True)

calc = SpookyNetCalculator(
    load_from="parameters.pth",
    charge=0,
    magmom=0
)
atoms.calc = calc

output_dir = "data/Pt_OH_H2O_relax"
os.makedirs(output_dir, exist_ok=True)

output_traj = os.path.join(output_dir, "Pt-OH-H2O_optim.traj")

opt = BFGS(atoms, trajectory=output_traj)

opt.run(fmax=0.2, steps=1000)
```

Show hidden output

```
atoms = read('/content/SpookyNet/data/Pt_OH_H2O_relax/Pt-OH-H2O_optim.traj', index=-1)
view(atoms, viewer='x3d')
```

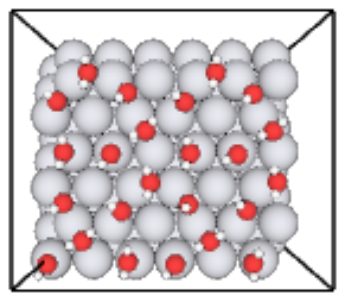

Conclusive remarks

In this tutorial, we started from a Pt(111) surface model containing an OH adsorbate and surrounding water molecules to represent a realistic catalytic interface. The structure was loaded into ASE and prepared for simulation by constraining the platinum atoms of the slab to mimic a bulk surface environment. Next, the SpookyNet machine-learning force field was attached as the calculator to predict energies and forces for the system. Using these ML-predicted forces, the interface was relaxed with a BFGS optimizer until the atomic configuration reached a stable geometry. This workflow demonstrates how ML force fields can be integrated into atomistic simulations of catalytic interfaces containing solvent molecules.

After completing this tutorial, you will be able to:

- Apply structural constraints to represent a realistic surface environment during simulations.
- Run geometry optimizations using a machine-learning force field through the SpookyNet framework.
- Integrate ML-based force fields into an ASE workflow for efficient relaxation of catalytic interfaces.

Tutorial 7. Machine-learning interatomic potentials for molecular dynamics

Goal of the notebook

This notebook demonstrates how molecular dynamics simulations of a catalytic interface can be performed using a machine-learning interatomic potential (MACE).

Description of the method

MACE (Message Passing Atomic Cluster Expansion) is a machine-learning interatomic potential trained on DFT data that predicts energies and forces for atomic systems. It combines cluster expansion physics with message-passing neural networks, allowing efficient simulations of large systems and long molecular-dynamics trajectories while maintaining near-DFT accuracy.

MACE github: https://github.com/ACEsuit/mace

MACE article: Batatia, I.; Kovács, D. P.; Simm, G. N. C.; Ortner, C.; Csányi, G. MACE: Higher Order Equivariant Message Passing Neural Networks for Fast and Accurate Force Fields. J. Chem. Phys. 2023. https://doi.org/10.48550/arXiv.2206.07697

Outline of theWorkflow

STEP 1. Preparing the Pt–OH–H_2O interface

STEP 2. Load the pretrained MACE potential and attach it as the calculator.

STEP 3. Run molecular dynamics simulations using ML-predicted forces.

STEP 4. Analyze the atomic trajectory to observe the dynamic behavior of water near the catalytic surface.

Installation of packages

First things first, we need to install the packages and import the neccessary functions. We need ASE, Torch and MACE

```
%%capture
!pip install mace-torch ase
```

Next we import the necessary packages:

ASE https://ase-lib.org/

MACE: https://github.com/ACEsuit/mace

Torch: https://github.com/pytorch/pytorch

```
from ase import units
from ase.constraints import FixAtoms
from ase.io import read, write, Trajectory
from ase.md.langevin import Langevin
from ase.units import fs
from ase.visualize import view
from mace.calculators import MACECalculator
from PIL import Image
import imageio
import torch
import os
from IPython.display import Image as IPImage
```

Show hidden output

STEP 1. Preparing the Pt–OH–H_2O interface

In this step we obtain the MACE calculator from GitHub and download the Pt(111)–OH–H_2O interface structure that will be used in the simulation. The structure is loaded into ASE and prepared as the starting configuration for the molecular dynamics simulation.

```
%%capture
!wget https://raw.githubusercontent.com/vilab-tartu/test_models/refs/heads/main/Pt-w/Pt-32H2O_MD.xyz
```

```
!wget https://github.com/ACEsuit/mace-mp/releases/download/mace_mp_0b/mace_agnesi_small.model
```

View the structure

```
method='mace'
prefix='Pt-32H2O_MD'
```

```
atoms = read(f'{prefix}.xyz')
view(atoms, viewer='x3d')
```

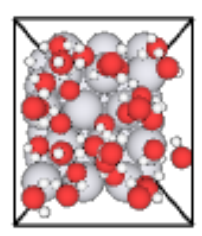

Fix all the atoms with tag = 1 (Pt)

```
pt_indices = [atom.index for atom in atoms if atom.tag == 1]
atoms.set_constraint(FixAtoms(indices=pt_indices))
```

STEP 2. Load the pretrained MACE potential and attach it as the calculator.

The pretrained MACE model is loaded and assigned as the calculator for the system. The model predicts energies and forces for the atomic configuration, replacing conventional DFT calculations during the simulation.

```
model_path = "mace_agnesi_small.model"
atoms.calc = MACECalculator(model_paths=model_path, use_cu_eq=False)
```

Show hidden output

STEP 3. Running molecular dynamics

Using the forces predicted by the MACE potential, a molecular dynamics simulation is performed. During the simulation the atomic positions evolve over time, allowing us to observe the movement of water molecules and their interactions with the catalytic surface.

```
dyn = Langevin(
    atoms,
    timestep=1.0 * fs,
    temperature_K=300.0,
    friction=0.01 / fs,
)

output_traj_file = f'{prefix}.traj'
traj = Trajectory(output_traj_file, 'w', atoms)
dyn.attach(traj.write, interval=1)

dyn.run(100)
```

STEP 4. Analyzing the trajectory

The generated trajectory is analyzed to examine the structure and dynamics of the interfacial water layer. This allows visualization of hydrogen bonding, adsorption stability, and the dynamic rearrangement of molecules near the Pt surface.

```
def trajectory_to_gif(traj_path, gif_name, rotation='-90x,90y,0z', show_unit_cell=0, duration=5.1):
    trajectory = read(traj_path, index=":")
    png_files = []

    # Write each frame as PNG
    for i, atoms in enumerate(trajectory):
        filename = f"frame_{i:04d}.png"
        write(filename, atoms, show_unit_cell=show_unit_cell, rotation=rotation)
        png_files.append(filename)

    # Prepare images with white background for GIF
    base_size = Image.open(png_files[0]).size
    images = []
    for fname in png_files:
        im = Image.open(fname).convert("RGBA")
        background = Image.new("RGBA", im.size, (255, 255, 255, 255))
        combined = Image.alpha_composite(background, im).convert("RGB")
        images.append(combined.resize(base_size))

    # Save GIF
    imageio.mimsave(gif_name, images, duration=duration)

    # Cleanup PNG files
    for fname in png_files:
        os.remove(fname)
    print(f"GIF saved as {gif_name}")

# Create GIF from trajectory
trajectory_to_gif(f'{prefix}.traj', f"{prefix}_{method}.gif")
```

Show hidden output

```
IPImage(f"{prefix}_{method}.gif")
```

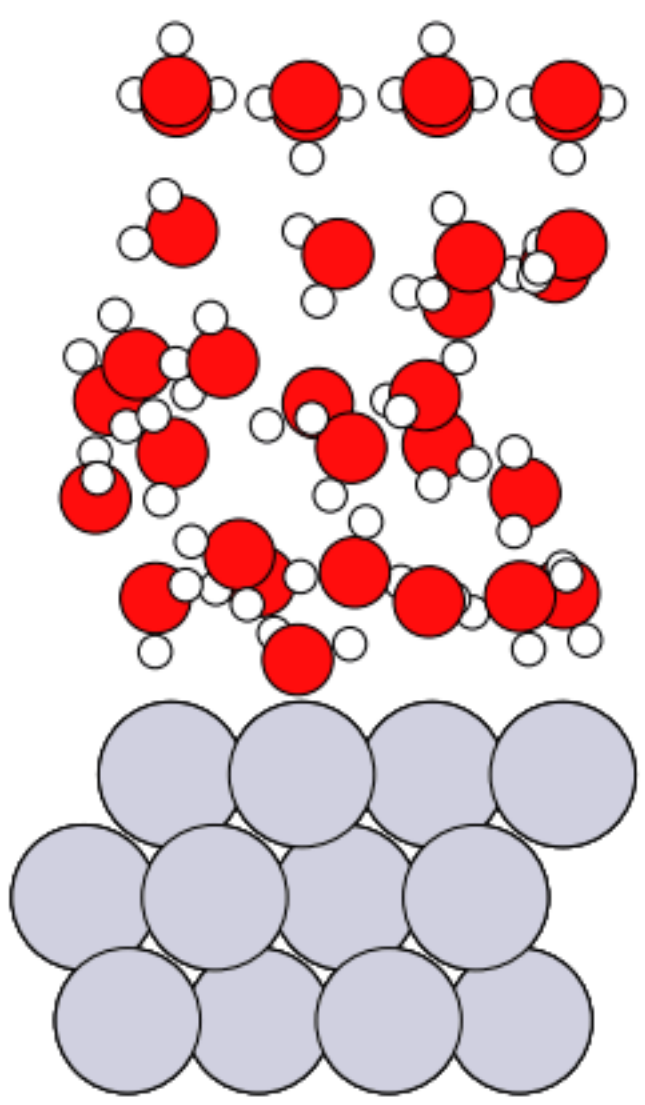

Conclusive remarks

In this tutorial, we started from a Pt(111)–OH–H_2O interface model representing a realistic electrochemical environment. The structure was loaded into ASE and the MACE machine-learning interatomic potential was attached as the calculator. Using ML-predicted energies and forces, a molecular dynamics simulation was performed to explore the time evolution of the system. The resulting trajectory illustrates how water molecules dynamically interact with the catalytic surface and the OH adsorbate.

After completing this tutorial, you will be able to:

- Perform molecular dynamics simulations of catalytic interfaces using machine-learning interatomic potentials.
- Load and apply pretrained MACE models within an ASE simulation workflow.
- Analyze trajectories from MD simulations to study the dynamic behavior of solvent molecules near catalytic surfaces.
- Use ML potentials to extend atomistic simulations to time scales that are difficult to access with conventional DFT methods.
